# A Novel Insulin/Glucose Model after a Mixed-Meal Test in Patients with Type 1 Diabetes on Insulin Pump Therapy

**DOI:** 10.1038/srep36029

**Published:** 2016-11-08

**Authors:** Luca Marchetti, Federico Reali, Marco Dauriz, Corinna Brangani, Linda Boselli, Giulia Ceradini, Enzo Bonora, Riccardo C. Bonadonna, Corrado Priami

**Affiliations:** 1 The Microsoft Research - University of Trento Centre for Computational and Systems Biology (COSBI), Rovereto (TN), Italy; 2 Department of Mathematics, University of Trento, Trento, Italy; 3 Department of Medicine, Section of Endocrinology, University of Verona School of Medicine, Verona, Italy; 4 Division of Endocrinology and Metabolic Diseases, Azienda Ospedaliera Universitaria Integrata, Verona, Italy; 5 Department of Clinical and Experimental Medicine, University of Parma, Parma, Italy; 6 Division of Endocrinology, Azienda Ospedaliera Universitaria of Parma, Italy

## Abstract

Current closed-loop insulin delivery methods stem from sophisticated models of the glucose-insulin (G/I) system, mostly based on complex studies employing glucose tracer technology. We tested the performance of a new minimal model (GLUKINSLOOP 2.0) of the G/I system to characterize the glucose and insulin dynamics during multiple mixed meal tests (MMT) of different sizes in patients with type 1 diabetes (T1D) on insulin pump therapy (continuous subcutaneous insulin infusion, CSII). The GLUKINSLOOP 2.0 identified the G/I system, provided a close fit of the G/I time-courses and showed acceptable reproducibility of the G/I system parameters in repeated studies of identical and double-sized MMTs. This model can provide a fairly good and reproducible description of the G/I system in T1D patients on CSII, and it may be applied to create a bank of “virtual” patients. Our results might be relevant at improving the architecture of upcoming closed-loop CSII systems.

The glucose-insulin (G/I) system is a physiological closed-loop, which is able to maintain the plasma glucose levels within a narrow physiological range, as a result of a complex interaction among many components^1^,^2^. Of them, only a limited number (namely, plasma glucose, insulin and C-peptide levels) is directly accessible for measurement in the bloodstream. Thus, either the closed-loop is experimentally interrupted under strictly controlled conditions, i.e. by the glucose clamp technique^3^, or dedicated mathematical models^4^,^5^ are needed to estimate the intimate components of the G/I system^6^,^7^,^8^,^9^,^10^,^11^,^12^,^13^,^14^,^15^,^16^,^17^,^18^,^19^,^20^,^21^,^22^.

Over the past four decades a number of experimental protocols have been developed to assess the dynamics of the G/I system *in vivo*^3^,^6^,^7^,^12^,^15^,^20^,^23^,^24^, often analyzed by multi-compartmental modeling techniques^9^,^22^,^25^. In such models the G/I dynamics are described through a set of ordinary differential equations (ODEs) according to well validated modeling strategies, which often require complex experimental settings, including the use of a variable number of glucose tracers to exactly track the glucose dynamics^12^,^26^,^27^. In order to mitigate the burden of both experimental and modeling complexity, more parsimonious models, i.e. with a lower number of ODEs and of parameters, have been proposed and successfully employed^6^,^15^,^23^,^28^,^29^,^30^,^31^,^32^,^33^. These “minimal models” have been thus far applied most frequently to the intravenous glucose tolerance tests (IVGTT) with the primary aim of measuring insulin sensitivity^6^,^14^,^33^. Their extension to more physiological settings, such as oral glucose tolerance tests (OGTT)^17^,^23^ and mixed meal tests (MTT)^30^,^34^, although feasible and widely in use, relies, when no glucose tracer(s) is (are) used, on an additional number of assumptions, especially regarding the dynamics of oral glucose appearance into the peripheral circulation^31^, plus glucose effectiveness, volume of distribution^26^,^35^ and splanchnic extraction^31^,^36^. Although the insulin sensitivities yielded by the oral models are well correlated to those obtained by the IVGTTs, they may overestimate insulin sensitivity, as assessed by the IVGTT^26^,^28^,^31^,^37^. Furthermore, insulin sensitivity provided by the OGTT model is lower than insulin sensitivity measured by the insulin clamp^38^ and higher than insulin sensitivity estimated by the MTT model^39^. Current evidence, however, indicates that, when measured with appropriate tools, i.e. tracer aided models of glucose dynamics, insulin sensitivity is relatively constant, regardless of the route of glucose/carbohydrate entry in the body^26^,^38^. Thus, current minimal modeling of glucose/carbohydrate meals with no tracer(s) aid, even though calibrated to successfully handle the oral glucose rate of appearance with a set of constrained parameters^31^,^36^, provides estimates of insulin sensitivity which, albeit correlated to those obtained with reference methods, display significant deviations from all other methods for somewhat unclear reasons^26^,^28^,^31^,^37^,^38^,^39^.

Modeling the G/I system is particularly relevant nowadays in the therapeutic area of type 1 diabetes (T1D), specifically in those patients treated with continuous subcutaneous insulin infusions (CSII) coupled with continuous glucose monitoring (CGM). A considerable research effort has resulted in G/I models based on the results of complex tracer studies^40^,^41^ and growing experience has been accrued to successfully close the loop with control algorithms of the G/I system derived from them^7^,^42^. Recent real-life clinical trials have reported very promising results towards the development of a reliable, wearable closed-loop insulin delivery system^43^,^44^,^45^.

For the reasons described above, published parsimonious models may be of limited help for these specific applications. We reasoned that some limitations of the most parsimonious G/I models (e.g. inaccurate estimation of insulin sensitivity, multiple assumptions in key parameters of the G/I system) could be overcome by combining the assessment of insulin sensitivity yielded by a gold standard technique, i.e. the hyperinsulinemic euglycemic clamp (HEC), with minimal modeling previously applied by us to unlabeled IVGTTs^46^ with slight modifications inspired by our experience with labeled IVGTT^22^.

This paper presents a novel mathematical model (GLUKINSLOOP 2.0) aimed at characterizing the G/I time-courses and quantitating the components of the G/I system during a standardized meal test. The GLUKINSLOOP 2.0 model builds on previous experience in our lab^22^,^46^ and provides a comprehensive description of the G/I system by introducing an original solution to model glucose dynamics after meals, which is usually accommodated with either a piecewise linear continuous function, partial differential equations, or delay compartments, or a combination of them^16^,^17^,^28^,^31^.

Owing to the potential applicability of this work in the field of T1D, the GLUKINSLOOP 2.0 model has been used to describe the G/I system during a mixed meal test (MMT) in patients with T1D on insulin pump therapy. Among the ten patients considered, six were studied twice in separate days with MMTs of variable size in order to explore both the performance and the reproducibility of GLUKINSLOOP 2.0. Our results might be relevant to strategies aimed at improving the architecture of upcoming closed-loop insulin delivery systems.

## Results

**Figure 1:**
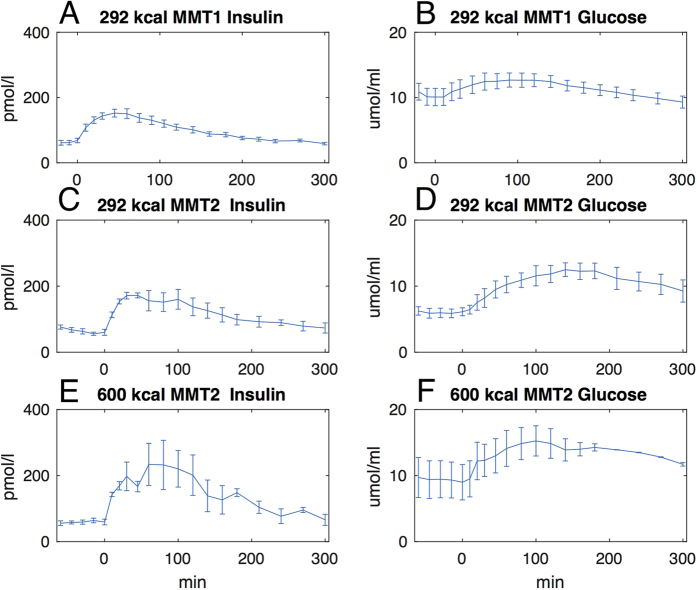
Time courses of plasma insulin and glucose levels during the 292 Kcal and 600 Kcal MMTs. Panels A,B: mean (±SEM) plasma insulin and glucose concentrations at each time point during the 292 Kcal MMT (*MMT1*) in the 10 study participants. Panels C,D: *MMT2*, n = 3, MMT = 292 Kcal. Panels E-F: *MMT2*, n = 3, MMT = 600 Kcal.

**Figure 2:**
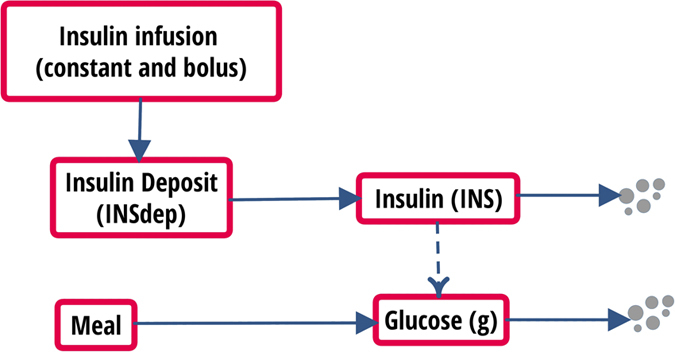
The GLUKINSLOOP 2.0 model. In this schematic representation^57^ of the model continuous arrows indicate transformations and dashed ones indicate regulations. Arrows pointing towards grey dots indicate degradation. A more detailed figure and an accompanying thorough explanation of the model are provided in the Supplementary Material (Figure S1).

The main clinical and metabolic features of the study patients are shown in Table 1. A quite large heterogeneity was evident in terms of age, body size, glucose control, duration of diabetes and time since the initiation of CSII therapy. When compared to historical healthy controls, the study patients had somewhat lower insulin sensitivity^47^. Figure 1 shows the time courses of plasma insulin and glucose concentrations during the 292 Kcal MTTs and during the 600 Kcal MTT, calculated as the average (±SEM) of the insulin and glucose concentrations at each time point during the MMTs for each group of patients undergoing the metabolic studies. As expected, plasma glucose/insulin time-series were higher in the latter (panels E-F) than in the former (panels A-D) set of MTTs. Figure 2 provides a simplified description of the GLUKINSLOOP 2.0 model herein applied to describe the G/I system and to identify its (unknown) parameters. The figure highlights the inherent conciseness of this new modeling solution, which is based on a parsimonious core set of ordinary differential equations (ODEs), as further detailed in the Methods section. A more detailed scheme and an accompanying thorough explanation of the GLUKINSLOOP 2.0 model equations are provided in the Supplementary Material (Figure S1 and Supplementary Note).

Visual inspection of weighted residuals indicates a good fit of the model to the experimental data (Fig. 3). The simulation outputs, expressed as model fits to the insulin and glucose curves, are provided in Figures S2-S11. In Figures S2-S7 the curves for each repeated study patient, during the 292 Kcal and 600 Kcal meals, are labeled as MMT1 and MMT2, respectively.

**Figure 4:**
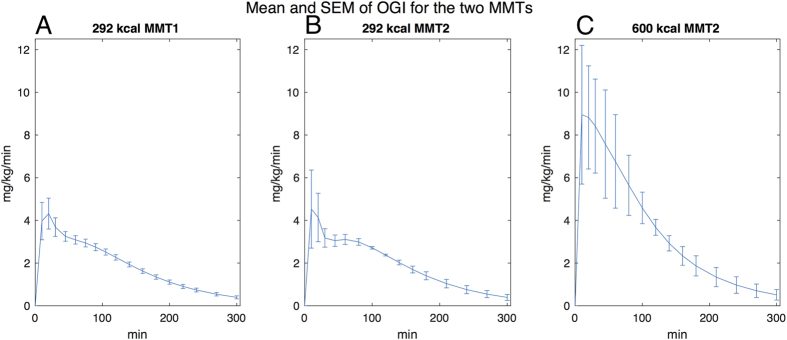
The Oral Glucose Input function. Panels (A–C) show the output of the OGI function layered by different MMTs. The OGI function, i.e. the predicted glucose rate of appearance in the bloodstream at each time point after the MMT ingestion, is provided as mean ± SEM of the individual OGI values for the study patients undergoing the MMT1 and MMT2 and is expressed as mg/Kg/min (see Figure S12 for the same panels expressed as μmol/min). Curves’ shapes and peaks are consistent with literature’s analogous functions^26^. Detailed description of the function can be found in Supplementary Material.

Figure 4 shows the mean behavior (mean ± SEM) of the Oral Glucose Input function (OGI), among the patients for the different meals. The shape and the peak of the curve agree with existing literature^26^.

Repeated MMTs showed a good degree of reproducibility of the key physiological parameters (Table 2 and Supplementary Table S4). Importantly, reproducibility was fairly good even when comparing meals of different sizes (Table 2 and Supplementary Table S4, patients 4, 5 and 6). Mean transit time of insulin (Insulin MTT) in the s.c. deposit were 112 ± 56 (min) for MMT1 and 131 ± 66 (min) for MMT2, respectively (Table 2); its within-subject coefficient of variation (±SD) was 28 ± 18% (Table 3). The apparent mean transit time of the oral glucose load from ingestion to the appearance in the accessible glucose pool (Glucose MTT) were 117 ± 35 (min) for MMT1 and 109 ± 35 (min) for MMT2, respectively (Table 2); its within-subject coefficient of variation (±SD) was 11 ± 6% (Table 3).

## Discussion

In this study, we successfully tested the hypothesis that, with the aid of the “external” assessment of insulin sensitivity by the HEC, the G/I system would be amenable to be successfully reconstructed in T1D patients, in whom modeling of the G/I system has become a key component of therapeutic innovative strategies^43^,^44^,^45^. Differently from previous models, which need to fix a number of parameters (glucose effectiveness, volume of distribution, fractional splanchnic extraction of glucose) to estimate meal insulin sensitivity, we exploit clamp-derived insulin sensitivity and parameters to reconstruct the G/I system during a mixed meal.

The novelty of our approach lies primarily in parsimony. Published models for OGTTs or meal tests were first based on more complex structures of the glucose system (typically two compartments were needed to accommodate glucose dynamics) and this, at variance with our proposal, entailed the need of tracer technology to identify the parameters governing the glucose system^7^,^8^,^9^,^12^,^26^,^48^. In the last 15 years, single compartment models were introduced, both without and with the aid of tracer technology^11^,^17^,^23^,^28^,^29^,^30^,^31^,^34^,^49^. Single compartment models with no tracer aid require a number of assumptions to reconstruct a reliable estimate of the rate of appearance of oral glucose, but they may provide somewhat variable estimates of insulin sensitivity^26^,^28^,^31^,^37^,^38^. The single compartment model with oral tracer glucose performs better than in the absence of a glucose tracer; however, its estimate of endogenous glucose production is good when expressed as the ratio of basal endogenous glucose production, but it may be inaccurate in absolute terms^35^. Increasing the number of tracers to two or three yields the best available estimates of endogenous glucose production, insulin sensitivity and glucose disposal, but it considerably increases study complexity and costs^27^.

Our approach transfers the single glucose compartment description of the time honored minimal model from the IVGTT^6^ to the MMT^7^. So far, a parsimonious description of the OGTT^23^,^30^ was focused only on the use of the OGTT as a test to assess insulin sensitivity^23^,^30^,^32^, at the cost of embodying a number of assumptions, of fixing numerical values for some parameters and of eventually providing insulin sensitivity values, which may be somewhat inaccurate.

These limitations are overcome by the herein presented GLUKINSLOOP 2.0 model of the G/I system. The performance of our model, however, does not contradict the extensive previous experience with the single compartment OGTT (and MTT) models with no glucose tracer(s)^23^,^28^,^30^,^31^,^32^. Early experience taught us that, when insulin sensitivity is unknown, it can be estimated from the oral tests at the cost of several approximations and assumptions in the parameters governing glucose dynamics^23^,^30^,^31^,^32^. Our present experience is logically coherent and complementary to the previous one, in that, if insulin sensitivity is known with the low uncertainty provided by the hyperinsulinemic euglycemic clamp (HEC), the key parameters of the G/I system can be safely estimated and a parsimonious description of the system can provide a good description of the glucose dynamics.

As an added value, this evidence is obtained in patients with T1D, in whom new, parsimonious models of the G/I system may improve current efforts in building algorithms capable to safely and precisely deliver insulin in the context of closed-loop devices^42^. From this viewpoint, our model has the attractive feature of showing a good degree of reproducibility of its key physiological parameters from day to day, and also with different meal sizes (Table 2). Of note, both the performance and robustness of the G/I dynamics estimates yielded by the GLUKINSLOOP 2.0 model were quite satisfactory despite the wide clinical heterogeneity of the study patients in terms of age, sex, diabetes duration and glycemic control.

Some specific characteristics of our model are different from the original minimal models and need to be discussed in some detail. First, in contrast with some previous single-compartment models with no tracer(s), we explicitly deal with glucose fluxes, and not concentrations^32^. The latter ones may be more convenient to handle, at least for the sake of simplicity. For instance, we are forced to know exactly the amount of glucose equivalents ingested. On the other hand, working with extensive properties of the G/I system (fluxes, volumes) is unavoidable, if one wants to obtain a complete description of the system and of its capability to cope with meal challenges, as well as to predict its behavior in response to meals of different size. Second, we introduced a fixed flux of glucose utilization, which primarily reflects brain glucose utilization, in agreement with the two-compartment minimal models proposed for studies with glucose tracer^22^,^25^, but not for single compartment models, such as the present one. In our opinion, the assumption of fixed glucose utilization (by the brain) is a perfectly tenable assumption and it is one of the improvements brought about by two-compartment minimal models for tracer glucose^25^ over the minimal models for unlabeled glucose^50^. In our experience, there is no reason why this improvement in the description of the system should not be implemented also in the models for unlabeled glucose. Third, the combined presence of a fixed glucose utilization and glucose effectiveness (S_G_) forced the need of a glucose input in the basal, un-stimulated state, which exactly matches the combined effect of brain glucose utilization and S_G_ and ensures the attainment of a steady state at baseline. Although it would be tempting to label this glucose input as endogenous glucose production - and its order of magnitude in our patients is indeed in the expected range - no measure of endogenous glucose production is available in our work and this glucose input should be considered as instrumental to the attainment of a steady state in the post-absorptive state. Fourth, as in all minimal models in which no glucose tracers are used, insulin sensitivity combines the net effect of insulin on glucose utilization and on glucose production^6^,^28^,^30^,^32^. Fifth, since no explicit endogenous glucose production is included in our model, the glucose input after meal ingestion should not be considered as a pure estimate of the appearance of oral glucose, because we cannot rule out the possibility that a minor fraction of it actually is due to an amount of residual endogenous glucose production, which is not captured by the insulin sensitivity parameter.

Some limitations of our study should be recognized. First, only patients with T1D are presented in this study; hence, the performance of our approach in normal individuals or in other pathologic conditions currently remains unexplored. However, the class of patients included in this study is expected to directly benefit most from novel simplified glucose models. Secondly, the number of studies herein presented is somewhat limited; however, this paper primarily aims at presenting the new GLUKINSLOOP 2.0 model, and it is not concerned with the report of novel pathophysiological insights. To this regard, it should be noted that the presentation of repeat studies with the same modeling methodology is a somewhat rare occurrence in this field and, as such, accounts for the stringent sensitivity analysis we applied to our data. Third, a separate insulin clamp (HEC) needs be performed to measure insulin sensitivity, thereby adding to the experimental burden one additional study day.

In summary, we have introduced a HEC-supported minimal model of glucose dynamics after a mixed meal in patients with T1D. The GLUKINSLOOP 2.0 model apparently performs reasonably well and shows a good degree of reproducibility without employing labeled tracers. Hence, given the relevance of *in vivo* characterization of the G/I system dynamics, this model timely proposes itself as a useful step towards better algorithms to control glucose dynamics after meal ingestion in patients with T1D on sensor-augmented insulin pump therapy.

## Methods

### Subjects

Ten (7 men/3 women) adult patients with C-peptide negative type 1 diabetes (T1D) on insulin pump therapy were recruited for the study among those regularly attending the Diabetes Center of the Verona City Hospital. Eligible patients included those 18–65 years old, on insulin pump therapy for at least 1 year, with HbA1c between 6.0 and 9.0%. Other inclusion/exclusion criteria can be found at the ClinicalTrials.gov website (https://clinicaltrials.gov/ct2/show/NCT02003274?term=NCT02003274&rank=1). Their main clinical characteristics are shown in Table 1. All patients were on isocaloric dietitian prescribed diet and were free from any other disease except diabetes (only Patient #3 had autoimmune hypothyroidism and was euthyroid on 150 μg/day levothyroxine p.o. at the time of study enrollment). After a thorough explanation of the procedures and purposes of the study, a written informed consent was obtained from all patients to be included in the study. The study protocol (registered as NCT01800734 on February 8^th^, 2013 and NCT02003274 on December 3^rd^, 2013) was approved by the local Institutional Review Board (Comitato etico per la sperimentazione clinica delle Provincie di Verona e Rovigo) and was carried out according to the International Conference on Harmonisation Good Clinical Practice guidelines.

### Phenotyping

Standard clinical parameters were assessed in all study patients. Metabolic tests were carried out at the Division of Endocrinology, Diabetes and Metabolism of the University of Verona Medical School (Verona, Italy), on three separate days, each test starting at 08:00 a.m., after a 10–12-h overnight fast. All patients were on CGM; the device had been in place and properly working (calibration with capillary blood glucose as measured by glucometer at pre-established hours of the day) for at least two days before metabolic studies. CGM data were collected for a companion experiment. During the entire study duration patients lay recumbent in bed. Two of the three studies were performed in random order. Study 1 - On one day, all patients underwent a hyperinsulinemic euglycemic insulin clamp (HEC). Study 2 - On a second occasion, all patients were studied with a standardized mixed meal test (MMT1) of 292 Kcal. Study 3 - The third study (MMT2) was in 3 patients the repetition of the 292 Kcal MMT, whereas in other 3 patients it consisted of a 600 Kcal MMT, with the same relative macronutrient composition of the 292 Kcal MTT.

### Assessment of Insulin Sensitivity (Study 1)

A standard HEC was carried out to assess insulin sensitivity, which was computed with standard formulae^3^,^51^, and expressed as the amount of glucose metabolized during the last 60 min of the clamp.

Subjects were instructed to use their usual nocturnal fast insulin analogue basal rate, to be left unchanged for at least five hours before the beginning of the test. Human insulin concentration was raised with an intravenous prime (0.8 U/m^2^ BSA) and maintained constant by a constant intravenous infusion (40 mU/min·m^2^ BSA). Plasma glucose was allowed to fall until it reached the physiologic range (i.e. <5.6 mmol/l), after which it was clamped at 5.0 mmol/l for at least 60 min by appropriately changing an intravenous infusion of 20% dextrose.

### Mixed-Meal Tests (Study 2 and 3)

The MMTs were performed to determine the time courses of plasma glucose and insulin during a mixed meal and to assess the pathophysiology of glucose control during a standardized physiological challenge. Subjects were instructed to be on an Indian corn free and cane sugar free diet for at least one week before each study and were instructed to use their usual nocturnal fast insulin analogue basal rate, to be left unchanged for at least five hours before the beginning of the test. A standardized mixed meal of maize polenta plus seasoned Italian Parmesan cheese (292 Kcal, 38.9 g carbohydrates, 8.9 g fats and 14 g proteins) was ingested by all study participants, and patients were monitored for 300 minutes thereafter. The time taken by the patients to ingest the meal was recorded. Right before meal ingestion, a fast subcutaneous insulin analogue bolus was administered through the pump, according to the individual insulin-to-carbohydrate ratio and correction dose.

On a separate day, a MMT of the same size was repeated in three patients, while a MMT with the same composition, but of double caloric size, was administered in the other three patients. In both cases, the experimental procedures were identical to the first MMT.

In all studies, blood samples were drawn at time intervals, put in ice and quickly spun at 1500 g at +4 °C. Plasma/serum specimens were stored at −80 °C.

### Measurements

Plasma glucose was measured in duplicate with an YSI 2300 Stat Plus Glucose&Lactate Analyzer (YSI Inc., Yellow Springs, OH, USA), at bedside. Blood samples were collected at timed intervals to measure plasma insulin. Interstitial glucose monitoring was performed by the CGM device throughout the entire duration of insulin clamp for a companion experiment. Serum insulin concentrations were measured by ELISA (Mercodia, Sweden)^51^, which can detect also lispro, aspart and glulisine insulin. Standard curves for each analog were used to measure accurately circulating insulin levels. To calculate the total insulin concentration achieved during the HEC, it was assumed that the insulin analog infused through the pump would generate the same concentration observed at baseline also throughout the entire clamp procedure. Glycosylated hemoglobin was measured by standard in-house methods. GAD-65 antibodies were measured by immunoradiometry (CentAK, Medipan, Germany), according to manufacturer’s instructions (detection limit >1 KU/l).

### Models

The MMT experiment, described in detail above, is modeled starting from the Minimal Model^6^,^50^ ideas and its further refinements^7^,^22^,^25^,^28^,^31^,^33^,^49^,^50^. Figure 2 and Figure S1 present a schematic representation of the model, which is tailored to consider T1D patients’ conditions and presents an original and physiologically plausible function, called Oral Glucose Input (OGI), to model the glucose appearance in the plasma. The MMT model is used to reproduce the insulin-glucose time series obtained during two different MMT experiments, as explained in Mixed-Meal Tests (Study 2 and 3). Parameters are estimated by fitting experimental data using *non-linear least squares* and a multi-start approach to ensure a global optimum. To reduce the uncertainty of parameter estimates, a combination of clamp-derived, patient-specific and literature-based prior information have been considered to drive the optimization process (see Technologies section and Supplementary Material). The robustness of the model has been also confirmed by the good reproducibility of parameter estimates on the two MMT experiments for all the physiological parameters (Table 2).

To simplify the description, the MMT model has been conceptually designed as being composed by two submodels (the “insulin” and the “glucose” submodel), which interact as shown in Figure S1. Since we are dealing here with T1D patients, the insulin submodel has been developed as a mono-compartmental model, where the beta-cell contribution to insulin secretion^15^,^20^,^52^,^53^ is removed. It describes the dynamics of the insulin deposit in tissues, due to the insulin injection, and the insulin concentration in the volume where insulin sampling takes place.

The glucose submodel is realized through a mono-compartmental model as well, where insulin action regulates glucose metabolism according to the minimal model principles^6^,^7^. The appearance of the ingested glucose in the system is obtained through the Oral Glucose Input function (OGI, see Supplementary Material). Such a function is the output of two chains of compartments, representing fast and slow glucose absorption during the digestion, which produces exponential-shaped outputs combined into a one/two peak(s) shape with exponential decay, as depicted in Fig. 4. This function integrates and extends previous observations^17^,^31^,^54^, by modeling the processes of digestion/absorption with just three parameters.

For a more detailed description of the model, we refer to the Supplementary Material. We refer to the Technologies section for further details on the implementation and parameter estimation procedures.

### Technologies

We implemented the GLUKINSLOOP 2.0 model in MATLAB v. R2016a (The MathWorks Inc., Natick, MA, USA) using ordinary differential equations (ODEs) simulated according to a Runge-Kutta algorithm. Model equations include sixteen unknown parameters. We carried out parameter estimation by *non-linear least squares* using the *lsqnonlin* function (MATLAB Optimization Toolbox v. R2016a, *trust-region-reflective* algorithm^55^,^56^) with a 1e-10 tolerance and a multi-start approach to ensure a global optimum (MATLAB Global Optimization Toolbox v. R2016a). To take into account the differences in concentration of insulin and glucose without introducing a bias in the fitting procedure, squared-relative-errors weighted on experimental standard deviations have been considered for parameter estimation. In addition, for each parameter whose prior information was available, a penalty term proportional to the distance of the current parameter estimate from the prior has been added to drive the optimization process.

## Additional Information

**How to cite this article**: Marchetti, L. *et al*. A Novel Insulin/Glucose Model after a Mixed-Meal Test in Patients with Type 1 Diabetes on Insulin Pump Therapy. *Sci. Rep*. **6**, 36029; doi: 10.1038/srep36029 (2016).

**Publisher’s note:** Springer Nature remains neutral with regard to jurisdictional claims in published maps and institutional affiliations.

## Supplementary Material

Supplementary Information

## Figures and Tables

**Figure 3:**
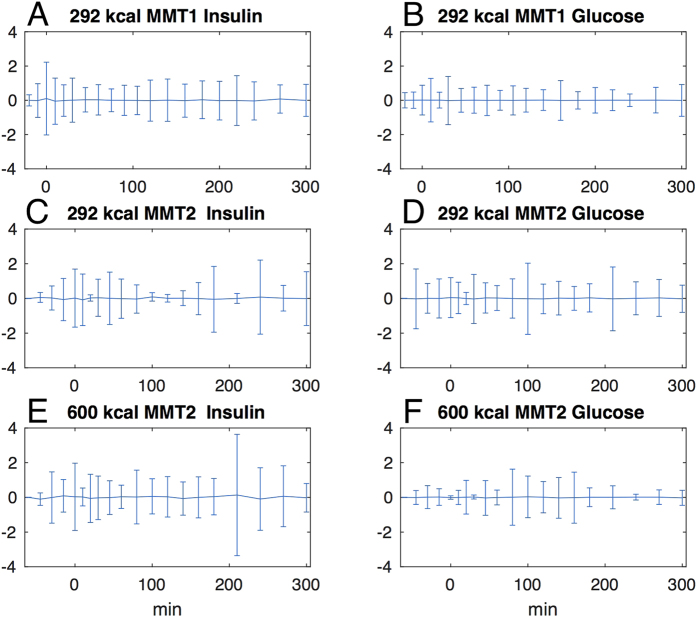
Mean weighted residuals of the model fit to experimental insulin and glucose time courses during MMT1 and MMT2. The weighted residuals are a quantitative point-by-point assessment of the goodness-of-fit of the model to the experimental data: a theoretically perfect fit should generate weighted residuals with mean 0 and SD of 1, reflecting the distribution of errors during the experimental sampling. Panels A,B: mean ± SD of weighted residuals at each time point during the 292 Kcal MMT (*MMT1*) in all 10 study participants. Panels C,D: *MMT2*, n = 3, MMT = 292 Kcal. Panels E,F: *MMT2*, n = 3, MMT = 600 Kcal.

**Table 1:** Clinical and metabolic features of the MMT-T1D Pilot Study population sample.

Variable	Patient 1	Patient 2	Patient 3	Patient 4	Patient 5	Patient 6	Patient 7	Patient 8	Patient 9	Patient 10
Sex	M	F	F	M	M	M	M	M	F	M
Age (years)	63	44	53	43	24	37	47	32	31	25
BMI (Kg·m^−2^)	26.43	20.60	24.75	19.27	24.73	25.54	23.29	23.27	21.80	22.89
BSA (m^2^)	1.91	1.47	1.63	1.72	1.80	1.97	1.99	1.89	1.73	1.83
HbA_1c DCCT_ (%)	8.0	8.3	7.1	7.2	7.8	8.7	8.6	7.3	7.8	7.2
HbA_1c IFCC_ (mmol/mol)	63.9	67.2	54.1	55.2	61.7	71.6	70.5	56.3	61.7	55.2
Duration of diabetes (years)	9	18	12	34	8	24	40	19	22	13
Duration of CSII therapy (years)	4	11	7	1	3	9	4	2	3	1
Insulin Sensitivity (M clamp) (μmol/min/m^2^ BSA)	1655	649	1272	830	829	1152	719	825	1861	965
Insulin analogue	aspart	lispro	aspart	glulisine	aspart	aspart	glulisine	aspart	lispro	lispro
MMT1 (292 Kcal)	•	•	•	•	•	•	•	•	•	•
MMT2 (292 Kcal)	•	•	•	—	—	—	—	—	—	—
MMT2 (600 Kcal)	—	—	—	•	•	•	—	—	—	—

BMI, Body Mass Index; BSA, Body Surface Area; HbA_1c DCCT_, Diabetes Control and Complication Trial-Aligned Hemoglobin A_1c_; HbA_1c IFCC_, International Federation of Clinical Chemistry-Aligned Hemoglobin A_1c_; CSII, Continuous Subcutaneous Insulin Infusion; MMT, Mixed Meal Test.

**Table 2:** Model estimates (mean ± SD) in all study participants of the key physiological parameters included in the GLUKINSLOOP 2.0.

Parameters	MMT1	MMT2
S_I,_ (ml/min)/(pmol/l)	0.78 ± 0.31	0.76 ± 0.38
S_G,_ ml/min	20.8 ± 18.4	24.2 ± 14.7
Glucose MTT, min	117 ± 35	109 ± 35
Insulin MTT, min	112 ± 36	131 ± 66

Data are provided as the average of parameter estimates (±SD) obtained by the model in all study participants during MMT1 and MMT2. MMT1, first mixed meal test; MMT2 second mixed meal test; S_I_, insulin sensitivity index; S_G_, glucose effectiveness; Glucose MTT, glucose mean transit time (gut-to-bloodstream) due to meal digestion; Insulin MTT, insulin mean transit time (subcutaneous depot-to-bloodstream). A complete description of the whole set of model parameters and their estimates in each study participant are provided in the Supplementary Material.

**Table 3:** Day-to-Day within-subject coefficients of variation (%; mean ± SD) of the key physiological parameters included in the GLUKINSLOOP 2.0.

Parameters	Coefficient of Variation (%; mean ± SD)
S_I_	12 ± 17
S_G_	23 ± 26
Glucose MTT	11 ± 6
Insulin MTT	28 ± 18

Only subjects 1-6, for which two studies are available, have been considered.
