# Supplementary material for: A Novel Insulin/Glucose Model after a Mixed-Meal Test in Patients with Type 1 Diabetes on Insulin Pump Therapy
Source: Sci Rep. 2016 Nov 8;6:36029. doi: 10.1038/srep36029 (PMC5099899; doi:10.1038/srep36029)
Supplement: Supplementary Information [file srep36029-s1.doc]

**Supplementary Material to:**

SREP-16-08101A **A Novel Insulin/Glucose Model after a Mixed-Meal Test in Patients with Type 1 Diabetes on Insulin Pump Therapy**

**Running title:** Computational modeling of CSII in MMT.

**Authors:** Luca Marchetti^1, §^, Federico Reali^1,2, §^, Marco Dauriz^3^, Corinna Brangani^3^, Linda Boselli^3^, Giulia Ceradini^3^, Enzo Bonora^3,4^, Riccardo C. Bonadonna^5,6*^ & Corrado Priami^1,2^

^§^ These Authors equally contributed to this work

^*^ Corresponding author

**Institutions**:

^1^ The Microsoft Research - University of Trento Centre for Computational and Systems Biology (COSBI), Rovereto (TN), Italy

^2^ Department of Mathematics, University of Trento, Trento, Italy

^3^ Department of Medicine, Section of Endocrinology, University of Verona School of Medicine, Verona, Italy

^4^ Division of Endocrinology and Metabolic Diseases, Azienda Ospedaliera Universitaria Integrata, Verona, Italy

^5^ Department of Clinical and Experimental Medicine, University of Parma, Parma, Italy

^6^ Division of Endocrinology, Azienda Ospedaliera Universitaria of Parma, Italy

## 1. Supplementary Material

**1.1 Supplementary Note pages 3-10**

**1.2 Supplementary Tables pages 11-16**

**Table S1: Unknown parameters of the Insulin Submodel.**

**Table S2: Unknown parameters of the Glucose Submodel.**

**Table S3: Unknown parameters of the HEC Submodel.**

**Table S4: Estimates of key physiological parameters of the GLUKINSLOOP 2.0 model during MMTs in each study participant.**

**Table S5: Estimates of the other parameters of GLUKINSLOOP 2.0 model during MMTs in each study participant.**

**Table S6: Abbreviations and acronyms.**

**1.3 Supplementary Figures pages 17-28**

**Figure S1: The GLUKINSLOOP 2.0 model.**

**Figures S2-S11: Simulation outputs of the GLUKINSLOOP 2.0 model for each study participant.**

**Figure S12: The Oral Glucose Input function (OGI).**

**1.4 References page 29**

**1.1 Supplementary Note**

**Models and Equations**

The GLUKINSLOOP 2.0 model herein presented has been developed as a set of ordinary differential equations (ODEs), which describe the regulation of glucose and insulin during a Mixed Meal Test (MMT) in T1D patients on insulin pump therapy (CSII, continuous subcutaneous insulin infusion). The GLUKINSLOOP 2.0 model is designed to run as a whole during simulation and parameter estimation. However, for the sake of simplicity and only for descriptive purposes, the model was conceptually divided in two submodels: the “insulin” and the “glucose” submodel.

The relationships among the compartments comprised in the GLUKINSLOOP 2.0 model and within its submodels are depicted in **Figure S1**.

The unknown parameters of the “insulin” and “glucose” submodels included in the ODEs (**Tables S1-S2**) have been estimated in each subject (**Tables S4-S5**) by fitting simultaneously the experimental time series of both circulating insulin and glucose (**Figures S2-S11**).

Section 1.1.4 provides the formal description of the model employed to fit the Hyperinsulinemic Euglycemic Clamp (HEC) experiment. The parameters estimates of the HEC model (**Table S3**) are entered in the GLUKINSLOOP 2.0 model (specifically, in the “glucose” submodel) to drive the estimation of a number of unknown parameters according to the following description.

**1.1.1 Mixed Meal (Insulin Submodel)**

The GLUKINSLOOP 2.0 is built on the assumption that insulin is provided through exogenous injection, since endogenous insulin secretion is not detectable in T1D. The Insulin Submodel (**Figure S1**, purple box) aims at fitting the time course of circulating insulin concentration during the MMT.

The Insulin Submodel is comprised of two equations. The first equation defines the INS_dep_ (µl of a 100 U/ml insulin solution) function, which represents the insulin deposit in tissues due to the exogenous CSII-driven insulin injection:


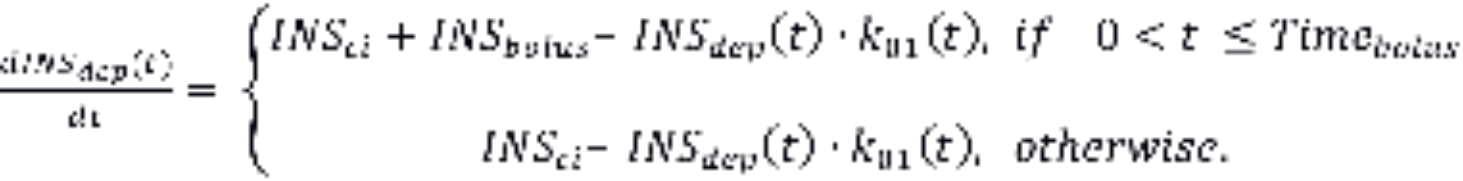
 (Eq. 1)

In the equation above INS_ci_ represents the constant insulin infusion (µl^.^min^-1^ of a 100 U/ml solution), while INS_bolus_ represents the insulin bolus injected before the mixed meal. Insulin transit from tissues to the bloodstream is modelled by the non-linear function
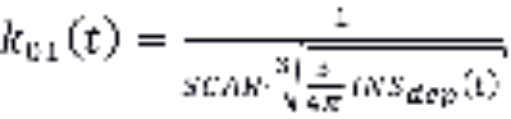
 which assumes that insulin diffusion is inversely proportional to the radius of a sphere with volume equal to INS_dep_ (SCAR is a parameter regulating insulin exit from the subcutaneous depot).

The second equation of the Insulin Submodel defines the INS(t) (pmol/l) function, which provides the insulin concentration dynamics in the compartment where the sampling of circulating insulin takes place:


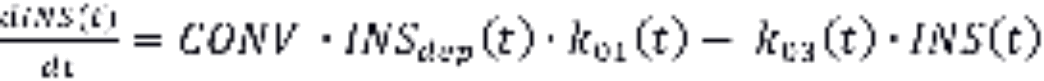
, (Eq. 2)

where CONV is a conversion factor between INS and INS_dep_, and
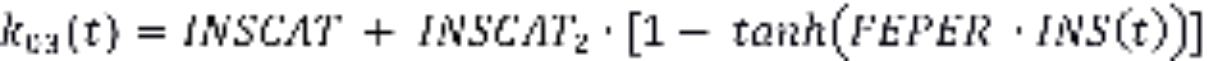
. The parameter k_03_(t) represents the time-varying clearance of insulin from the sampling compartment, in which the hyperbolic function describes the inverse relationship between insulin concentration and insulin clearance^1^ primarily due to nonlinearity and saturability of insulin extraction by the liver^2-4^.

The unknown parameters of the Insulin Submodel are summarized in **Table S1**. For each parameter an initial estimate is provided, along with the optimization boundaries assigned to the system to compute the final parameter estimate.

**1.1.2 Mixed Meal (Glucose Submodel)**

This submodel is related to glucose dynamics and is highlighted in skyblue in **Figure S1**.

The glucose input into the plasma compartment, due primarily to the ingestion of the mixed meal, is modelled by means of the Oral Glucose Input (OGI, μmol/min) function, described further below in Section 1.1.3.

Glucose dynamics, g (μmol), are described by the following equations:


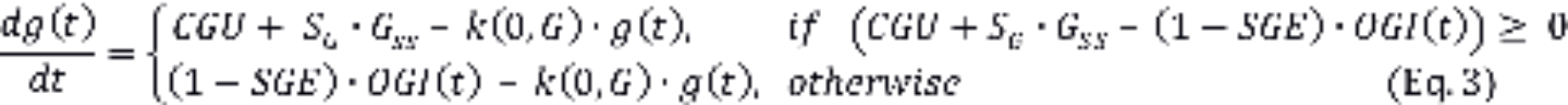


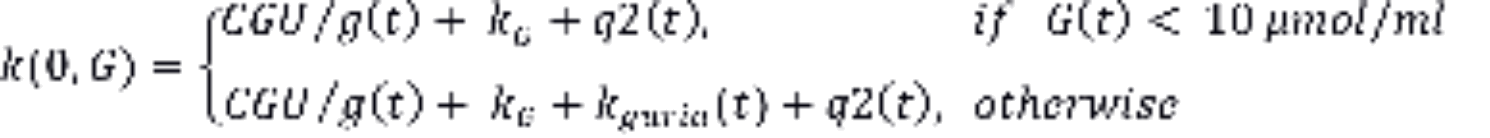
 (Eq. 4)

where SGE is the apparent fractional splanchnic glucose extraction, CGU is the constant glucose uptake, given by 203 **.** BSA (μmol/min); S_G_ is the glucose effectiveness, G_ss_ (μmol/ml) is the glucose concentration at the steady state (minimum value of the experimental data); G(t) is the glucose concentration (g(t)/V_G_) at time t; q2(t) indicates the insulin action at time t (see Eq. 5); and k_guria_(t) represents the time varying rate constant of glycosuria (min^-1^) derived from measured urinary glucose.

Formally:

*k_guria_(t) = [RClearance /g(t)]*(G(t)-10)* , where *RClearance = (Glycosuria) / {∫[(G(t)-10+|G(t)-10|)/2].dt}*

and the symbol ∫ indicates the integral function between the beginning and the end of the experiment. *RClearance* represents the renal clearance of glucose ((G_U_*V_U_)/G_P_, with G_U_ urinary glucose concentration; V_U_ urine volume; G_P_ plasma glucose level), which comes into play above the renal threshold of glycosuria (about 10 mmol/l).

The insulin action q2(t) (min^-1^) on glucose metabolism is described by the following equation:


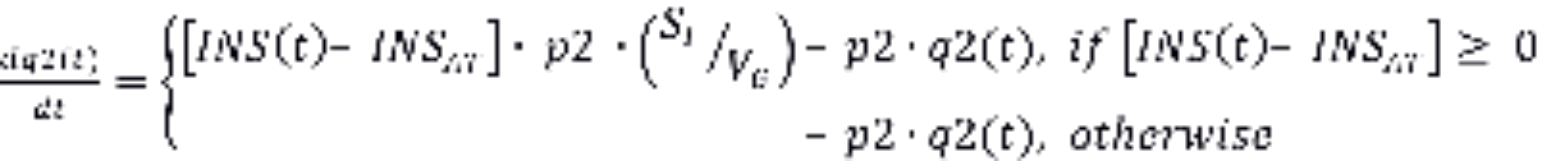
 (Eq. 5)

where INS(t) is the circulating insulin concentration at time t, computed by simulating the Insulin Submodel; INS_AT_ is the concentration threshold above which insulin action takes place; p2 is the rate constant of insulin action fading; V_G_ (milliliters, ml) is the apparent glucose distribution volume; and S_I_ is the insulin sensitivity at the steady state (see Section 1.1.4).

The unknown parameters of the Glucose Submodel are summarized in **Table S2**. For each parameter an initial estimate is provided, along with the optimization boundaries assigned to the system to compute the final parameter estimate. Of note, the Glucose Submodel includes four additional parameters (**Table S3**), estimated from the HEC experiment as detailed further below in **Section 1.1.4**. The HEC-derived parameter estimates drive the multistart fitting of the mixed meal model by initializing one starting point to the values computed during the HEC fitting. Moreover, the final estimates of V_G_, S_G_ and S_I_ have been assumed to follow a normal distribution of mean equal to the HEC estimate and standard deviation (SD) of 20%, which accounts for the variability of patient metabolism and uncertainties in experimental measures. This has been implemented by including a penalty term
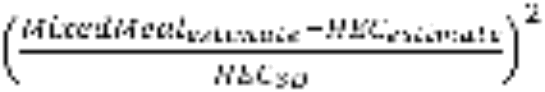
 in the objective function for each parameter. Also the estimation of the parameters INS_AT_ and SGE followed the same approach, where the mean of the INS_AT_ distribution has been determined by the insulin basal and the mean of the SGE distribution has been inferred from population data^5,6^.

**1.1.3 Oral Glucose Input (OGI) function**

The OGI(t) function describes the dynamics of glucose input (μmol/min) into the plasma compartment after the ingestion of the mixed meal and accounts for the delay of glucose appearance in the bloodstream after meal ingestion and gut transit (see **Figure S12**). The OGI function architecture rests on a multi-compartmental model constituted by two chains of 2 compartments (the minimum length required to build a delay chain):


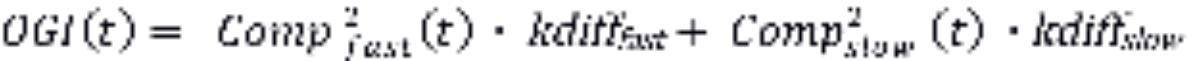
(Eq. 6)

where:

*[d Comp^2^_fast_ (t)/dt] = kdiff_fast_* ***.*** *(Comp^1^_fast_ (t) - Comp^2^_fast_ (t))*

*[d Comp^1^_fast_ (t)/dt] = k_fast_* ***.*** *Gload(t)- kdiff_fast_* ***.*** *Comp^1^_fast_ (t)*

*[d Comp^2^_slow_ (t)/dt] = kdiff_slow_* ***.*** *(Comp^1^_slow_ (t) – Comp^2^_slow_ (t))*

*[d Comp^1^_slow_ (t)/dt] = k_slow_* ***.*** *Gload(t)- kdiff_slow_* ***.*** *Comp^1^_slow_ (t)*

and:


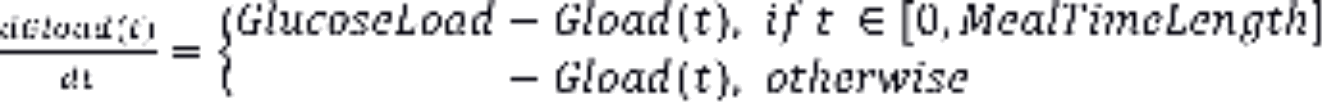
 (Eq. 7)

In the equations above, GlucoseLoad (μmol/min) represents the averaged glucose (carbohydrates) mass that is ingested in a minute during the meal, while k_fast_ and k_slow_ (min^-1^) are two kinetic parameters, representing the fraction of ingested glucose (carbohydrates) which follows the fast and slow route, respectively. As such, the net sum k_fast_ + k_slow_ = 1.

The kinetic parameters kdiff_fast_ and kdiff_slow_ (min^-1^) represent the rate constants at which glucose travels through each chain of compartments. The average time taken by glucose to travel through each chain of compartments is calculated as Ingested Glucose Transit Time (IGTT) of the fast and of the slow route, respectively (IGTT_fast_ and IGTT_slow_). Parameters kdiff_fast_ and kdiff_slow_ are computed as the number of compartments of the chain divided by the corresponding IGTT (2/IGTT_fast_ and 2/IGTT_slow_).

For each MMT, the Ingested Glucose Mean Transit Time (Glucose MTT1 and Glucose MMT2) provided in **Table II** of the main text and in **Table S4**, is computed by the weighted average of IGTT_fast_ and IGTT_slow_ according to their respective kinetic parameters, k_fast_ and k_slow_, as follows:*Glucose MTT = k_fast_*
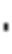
 *IGTT_fast_+ k_slow_*
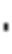
 *IGTT_slow_*

**1.1.4 Hyperinsulinemic Euglycemic Clamp (HEC Submodel)**

The MMT model introduced above has been coupled with a separate model describing the kinetics of glucose and insulin during the hyperinsulinemic euglycemic clamp (HEC) procedure in patients with diabetes, who are hyperglycemic in the fasting state. The HEC is the time-honoured gold standard to assess insulin sensitivity (S_I_)^7^. As explained above in Section 1.1.2, the estimates of the four parameters included in the model (V_G_, S_G_, p2 and S_I_) are employed in the GLUKINSLOOP 2.0 to drive the estimation of the corresponding parameters in the Glucose Submodel.

The kinetic of glucose and insulin during the HEC experiment are described by means of a mono-compartmental model (also referred as the “glucose metabolism” compartment), in which the dynamics of glucose (g) interact with the corresponding insulin action (q2) over time (t).

The glucose dynamics are described by the following equation:


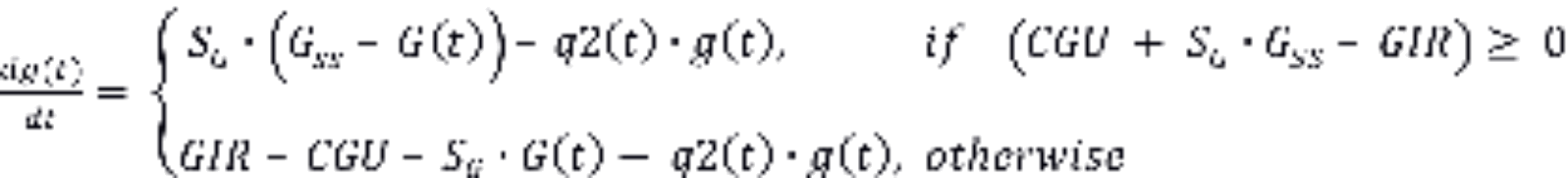
 (Eq. 8)

where CGU is the constant glucose uptake, given by 203 **.** BSA (μmol/min); S_G_ is the glucose effectiveness; G_ss_ is the glucose concentration at the steady state (i.e. the minimum value of the experimental time series); GIR is the intravenous glucose infusion rate; G(t) is the glucose concentration (g(t)/V_G_) at time t; and q2(t) is the insulin action at time t.

Insulin action q2(t) is described by the following equation:


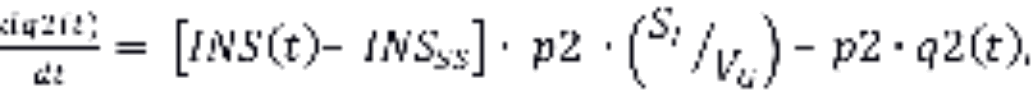
 (Eq. 9)

where INS(t) is the linear interpolation of insulin’s experimental data at time t, INS_SS_ is the insulin concentration at baseline, p2 is the rate constant of insulin action fading, V_G_ is the apparent glucose distribution volume and S_I_ is insulin sensitivity at steady state hyperinsulinemia.

In order to enhance the reliability of the estimation of S_I_, we refined its value starting from the initial estimate^8^:


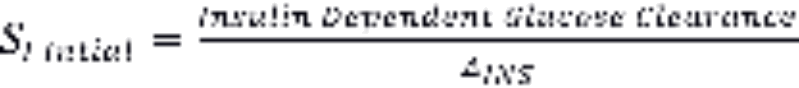
 , (Eq. 10)

where:

*Insulin Dependent Glucose Clearance = (M – CGU) / G_HECss_*

*Δ_INS_ = INS_HECss_ - INS_SS_*

The value of *M* (µmol^.^min^-1^) is the HEC-derived measure of whole-body insulin action and is defined as the average glucose infusion rate over the last 60 minutes of the HEC. G_HECss_ is the average glucose concentration at steady state of the HEC, INS_HECss_ is the average insulin concentration at steady state of the HEC and INS_SS_ is baseline insulin concentration.

The unknown parameters of the HEC Submodel are summarized in **Table S3**. For each parameter an initial estimate is provided, along with the optimization boundaries assigned to the system to compute the final parameter estimate.

**1.2 Supplementary Tables**

| **Table S1 – Unknown parameters of the Insulin Submodel.** | | | |
| --- | --- | --- | --- |
| **Parameter** | **Initial Value** | **Lower Bound** | **Upper Bound** |
| **I_dep_(0)** (µl) | 30 | 1 | 500 |
| **SCAR** (min(µl/min)^-1/3^) | 50 | 1 | 500 |
| **CONV** (unitless) | 20 | 1 | 1500 |
| **INSCAT** (min^-1^) | 0.05 | 0 | 10 |
| **INSCAT_2_** (min^-1^) | 0.05 | 0 | 10 |
| **FEPER** (pmol/l)^-1^ | 0.002 | 0.0001 | 0.05 |

| **Table S2 – Unknown parameters of the Glucose Submodel***. | | | |
| --- | --- | --- | --- |
| **Parameter** | **Initial Value** | **Lower Bound** | **Upper Bound** |
| **q2**(0) (min^-1^) | 0.001 | 0 | 0.05 |
| **SGE** (unitless) | 0.14 with 20% of SD ^5^ | 0 | - |
| **k_fast_** (min^-1^) | 0.3 | 0.01 | 1 |
| **GTT_fast_** (min) | 50 | 1 | 200 |
| **GTT_slow_** (min) | 80 | 10 | 300 |
| **INS_AT_** (pmol/l) | insulin basal with 20% of SD | 0 | - |

* parameter estimates from the HEC submodel are included according to **Section 1.1.2**

| **Table S3 – Unknown parameters of the HEC Submodel.** | | | |
| --- | --- | --- | --- |
| **Parameter** | **Initial Value** | **Lower Bound** | **Upper Bound** |
| **V_G_** (ml) | 10000 | 4000 | 20000 |
| **S_G_** (ml/min) | 40 | 0 | 250 |
| **p2** (min^-1^) | 0.1 | 0.0001 | 1 |
| **S_I_** ((ml/min)/(pmol/l)) | *S_I Initial_* (Eq. 10) | *S_I Initial_* -20% | *S_I Initial_* +20% |

| **Table S4 - Estimates of key physiological parameters of the GLUKINSLOOP 2.0 model during MMTs in each study participant.** S_I_: (ml/mol)/(pmol/l); S_G_: ml/min; MTT: min. | | | | | |
| --- | --- | --- | --- | --- | --- |
|  |  | **S_I_** | **S_G_** | **Ingested Glucose MTT** | **Subcutaneous Insulin MTT** |
| **Patient 1** | **MMT1** | 0.80 | 14.01 | 115 | 50 |
|  | **MMT2** | 0.80 | 31.11 | 107 | 108 |
| **Patient 2** | **MMT1** | 0.40 | 8.67 | 136 | 70 |
|  | **MMT2** | 0.42 | 17.66 | 152 | 77 |
| **Patient 3** | **MMT1** | 1.36 | 5.49E-13 | 144 | 178 |
|  | **MMT2** | 1.30 | 5.49E-13 | 87 | 130 |
| **Patient 4** | **MMT1** | 0.59 | 37.37 | 73 | 27 |
|  | **MMT2** | 0.29 | 46.28 | 62 | 53 |
| **Patient 5** | **MMT1** | 0.66 | 9.07 | 191 | 183 |
|  | **MMT2** | 0.67 | 9.29 | 147 | 221 |
| **Patient 6** | **MMT1** | 1.09 | 16.12 | 75 | 136 |
|  | **MMT2** | 1.08 | 16.49 | 99 | 196 |
| **Patient 7** | **MMT1** | 0.55 | 60.27 | 105 | 129 |
| **Patient 8** | **MMT1** | 0.61 | 27.70 | 92 | 170 |
| **Patient 9** | **MMT1** | 1.15 | 13.61 | 127 | 100 |
| **Patient 10** | **MMT1** | 0.60 | 4.54E-08 | 108 | 76 |

| **Table S5 – Estimates of the other parameters of GLUKINSLOOP 2.0 model during MMTs in each study participant (key physiological parameters are in Table S4).** V_G_: ml; p2: min^-1^; I_dep_0: µl; SCAR: min(µl/min)^-1/3^; CONV: unitless; INSCAT: min^-1^; INSCAT_2_: min^-1^; FEPER: (pmol/l)^-1^; q2(0): min^-1^; SGE: unitless; k_fast_: min^-1^; GTT_fast_: min; GTT_slow_: min; INS_AT_: pmol/l. | | | | | | | | | | | | | | | |
| --- | --- | --- | --- | --- | --- | --- | --- | --- | --- | --- | --- | --- | --- | --- | --- |
|  |  | **V_G_** | **p2** | **I_dep_0** | **SCAR** | **CONV** | **INSCAT** | **INSCAT_2_** | **FEPER** | **q2(0)** | **SGE** | **k_fast_** | **GTT_fast_** | **GTT_slow_** | **INS_AT_** |
| **Patient 1** | **MMT1** | 6317 | 0.00062 | 5.53 | 47.78 | 20.14 | 3.4E-02 | 3.4E-08 | 1.1E-04 | 1.1E-03 | 0.12 | 0.09 | 9 | 126 | 64.56 |
|  | **MMT2** | 7937 | 0.00182 | 25.51 | 106.36 | 66.93 | 1.1E-07 | 1.6E-01 | 3.1E-03 | 2.4E-14 | 0.14 | 0.16 | 12 | 125 | 66.08 |
| **Patient 2** | **MMT1** | 10994 | 0.99979 | 12.16 | 76.12 | 42.17 | 4.2E-03 | 1.4E-01 | 4.3E-03 | 1.1E-02 | 0.12 | 0.97 | 136 | 137 | 46.95 |
|  | **MMT2** | 8024 | 0.07434 | 21.08 | 83.77 | 183.30 | 1.5E-08 | 6.8E-01 | 4.3E-03 | 1.1E-02 | 0.12 | 0.07 | 37 | 160 | 55.57 |
| **Patient 3** | **MMT1** | 12225 | 0.00249 | 232.01 | 193.34 | 359.51 | 5.7E-01 | 2.3E+00 | 9.7E-03 | 1.9E-03 | 0.14 | 0.17 | 19 | 168 | 56.38 |
|  | **MMT2** | 12487 | 0.00113 | 60.15 | 133.19 | 166.30 | 1.7E-01 | 6.0E-01 | 8.7E-03 | 4.0E-14 | 0.14 | 0.18 | 11 | 103 | 59.65 |
| **Patient 4** | **MMT1** | 17250 | 0.00313 | 1.04 | 25.35 | 7.15 | 3.1E-07 | 2.3E-02 | 2.3E-03 | 3.4E-14 | 0.13 | 0.19 | 11 | 88 | 36.05 |
|  | **MMT2** | 20600 | 0.99473 | 10.45 | 40.57 | 20.11 | 8.6E-09 | 6.4E-02 | 1.6E-03 | 2.3E-02 | 0.13 | 0.06 | 3 | 66 | 50.80 |
| **Patient 5** | **MMT1** | 4290 | 0.00856 | 298.49 | 203.55 | 159.65 | 2.9E-04 | 1.3E+00 | 6.3E-03 | 1.7E-03 | 0.14 | 0.18 | 43 | 225 | 35.20 |
|  | **MMT2** | 6345 | 0.16826 | 414.32 | 203.69 | 677.68 | 1.6E+00 | 1.0E+01 | 1.3E-02 | 2.5E-14 | 0.14 | 0.15 | 30 | 168 | 47.34 |
| **Patient 6** | **MMT1** | 18874 | 0.00150 | 36.90 | 114.25 | 72.75 | 3.5E-07 | 3.1E-01 | 4.2E-03 | 1.2E-04 | 0.14 | 0.38 | 21 | 108 | 45.32 |
|  | **MMT2** | 21505 | 0.00059 | 111.36 | 172.93 | 267.20 | 2.4E-01 | 1.0E+00 | 6.7E-03 | 1.3E-03 | 0.14 | 0.24 | 17 | 125 | 63.88 |
| **Patient 7** | **MMT1** | 6353 | 0.00061 | 64.48 | 121.38 | 126.83 | 2.0E-01 | 8.7E-01 | 1.2E-02 | 6.2E-13 | 0.14 | 0.07 | 18 | 112 | 35.21 |
| **Patient 8** | **MMT1** | 16578 | 0.08556 | 383.92 | 177.62 | 200.92 | 2.6E-01 | 1.9E+00 | 7.2E-03 | 8.8E-03 | 0.13 | 0.21 | 24 | 111 | 70.81 |
| **Patient 9** | **MMT1** | 12587 | 0.09404 | 90.31 | 108.30 | 1312 | 1.3E+00 | 5.9E+00 | 6.1E-03 | 2.4E-14 | 0.12 | 0.06 | 4 | 134 | 63.52 |
| **Patient 10** | **MMT1** | 8160 | 0.01144 | 32.25 | 73.43 | 84.60 | 1.0E-01 | 3.5E-01 | 7.2E-03 | 2.5E-14 | 0.14 | 0.24 | 16 | 137 | 59.39 |

| **Table S6 – Abbreviations and acronyms.** | |
| --- | --- |
|  |  |
| BSA | Body Surface Area |
| CGM | Continuous Glucose Monitoring |
| CGU | Constant Glucose Uptake (μmol/min) |
| Comp_Fast_ | Fast route of ingested glucose from mouth to systemic circulation |
| Comp_Slow_ | Slow route of ingested glucose from mouth to systemic circulation |
| CONV | Scaling factor |
| CSII | Continuous Subcutaneous Insulin Infusion |
| FEPER | Parameter regulating nonlinear insulin clearance |
| g | Glucose mass (μmol) |
| G | Glucose concentration (μmol/l) |
| G_HECss_ | Average glucose concentration at steady state of the HEC |
| GIR | Intravenous Glucose Infusion Rate |
| G_P_ | Plasma glucose level |
| G_SS_ | Glucose concentration at the steady state |
| GTT_fast_ | Mean Transit Time of glucose in Comp_Fast_ |
| GTT_slow_ | Mean Transit Time of glucose in Comp_Slow_ |
| G_U_ | Urinary glucose concentration |
| HEC | Hyperinsulinemic Euglycemic Clamp |
| I_dep_(t) | Volume of Insulin Depot at time t |
| IGTT | Ingested Glucose Transit Time |
| INS | Plasma Insulin concentration |
| INS_AT_ | Insulin Action Threshold |
| INS_bolus_ | Insulin Bolus as infused by the insulin pump |
| INSCAT | Insulin Clearance (linear component) |
| INSCAT_2_ | Insulin Clearance (non-linear, saturable component) |
| INS_ci_ | Constant Insulin Infusion by the insulin pump |
| INS_dep_ | Subcutaneous Insulin Depot |
| INS_HECss_ | Average insulin concentration at steady state of the HEC |
| INS_SS_ | Insulin Concentration at baseline |
| IVGTT | Intravenous Glucose Tolerance Test |
| kdiff_fast_ | Rate constant of glucose through Comp_Fast_ |
| kdiff_slow_ | Rate constant of glucose through Comp_Slow_ |
| k_fast_ | Fraction of Ingested Glucose traveling through Comp_Fast_ |
| k_guria_(*t*) | Time varying rate constant of glycosuria |
| k_slow_ | Fraction of Ingested Glucose traveling through Comp_Slow_ |
| k_(m,n)_ | Kinetic parameters |
| M | HEC-derived measure of whole-body insulin action |
| MMT | Mixed Meal Test |
| MTT | Mean Transit Time |
| ODE | Ordinary Differential Equation |
| OGI | Oral Glucose Input Function |
| OGTT | Oral Glucose Tolerance Test |
| p2 | Rate Constant of Insulin Action Fading |
| q2(t) | Insulin Action at time t |
| RClearance | Renal Clearance of Plasma Glucose |
| SCAR | Parameter regulating insulin exit from the subcutaneous depot |
| S_G_ | Glucose Effectiveness |
| SD | Standard deviation |
| SGE | Splanchnic Glucose Extraction |
| S_I_ | Insulin Sensitivity at Steady State |
| T1D | Type 1 Diabetes |
| V_G_ | Apparent Glucose Volume of Distribution |
| V_U_ | Urine volume |

**1.3. Supplementary Figures**

**Figure S1 – The GLUKINSLOOP 2.0 model.** The continuous arrows indicate mass transfers while the dashed arrows connecting insulin to the insulin action compartment and then insulin action to the irreversible loss of the glucose compartment symbolize the control exerted by insulin on glucose metabolism. The arrows pointing toward grey dotted material are used to indicate irreversible losses. INS, plasma insulin concentration; INSci, constant insulin infusion by the insulin pump; INS bolus, insulin bolus infused by the insulin pump; INSdep, subcutaneous insulin deposit; CONV, scaling factor; k, kinetic parameters; t, time; CompFast1-2, fast route of ingested glucose from mouth to systemic circulation; CompSlow1-2, slow route of ingested glucose from mouth to systemic circulation; q2, insulin action; p2, rate constant of insulin action fading; SGE, Splanchnic Glucose Extraction; OGI, Oral Glucose Input function; g, glucose mass; G, glucose concentration; CGU, Constant Glucose Uptake; kG = S_G_/V_G_.


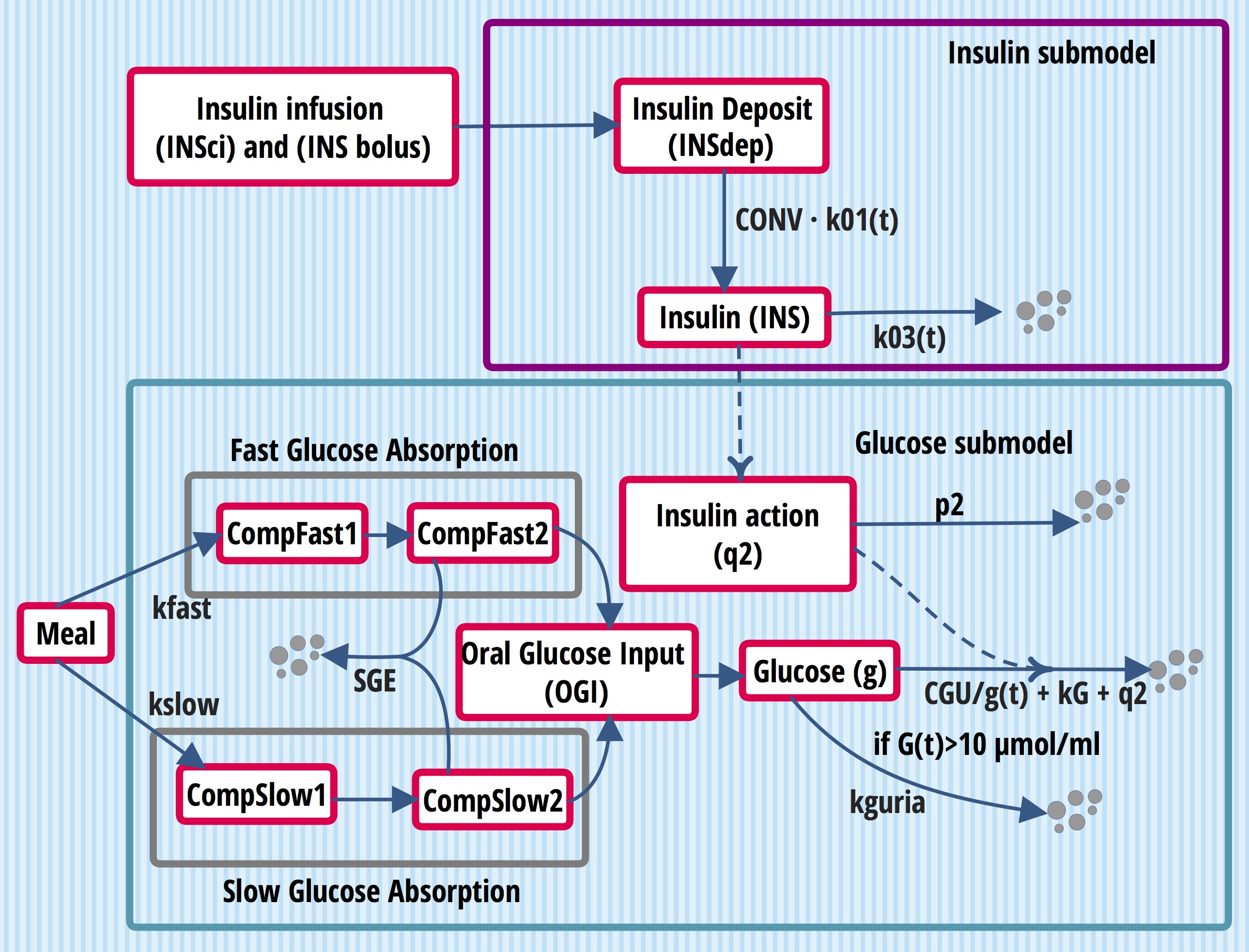


**Figure S2 - Simulation outputs of the GLUKINSLOOP 2.0 (patient 1).** The figure shows the time courses of plasma insulin and glucose concentrations during the MMT1 (left panel) and MMT2 (right panel) in the first study participant. Experimental data are shown as blue dots, while the simulated time courses are provided as a continuous blue line.


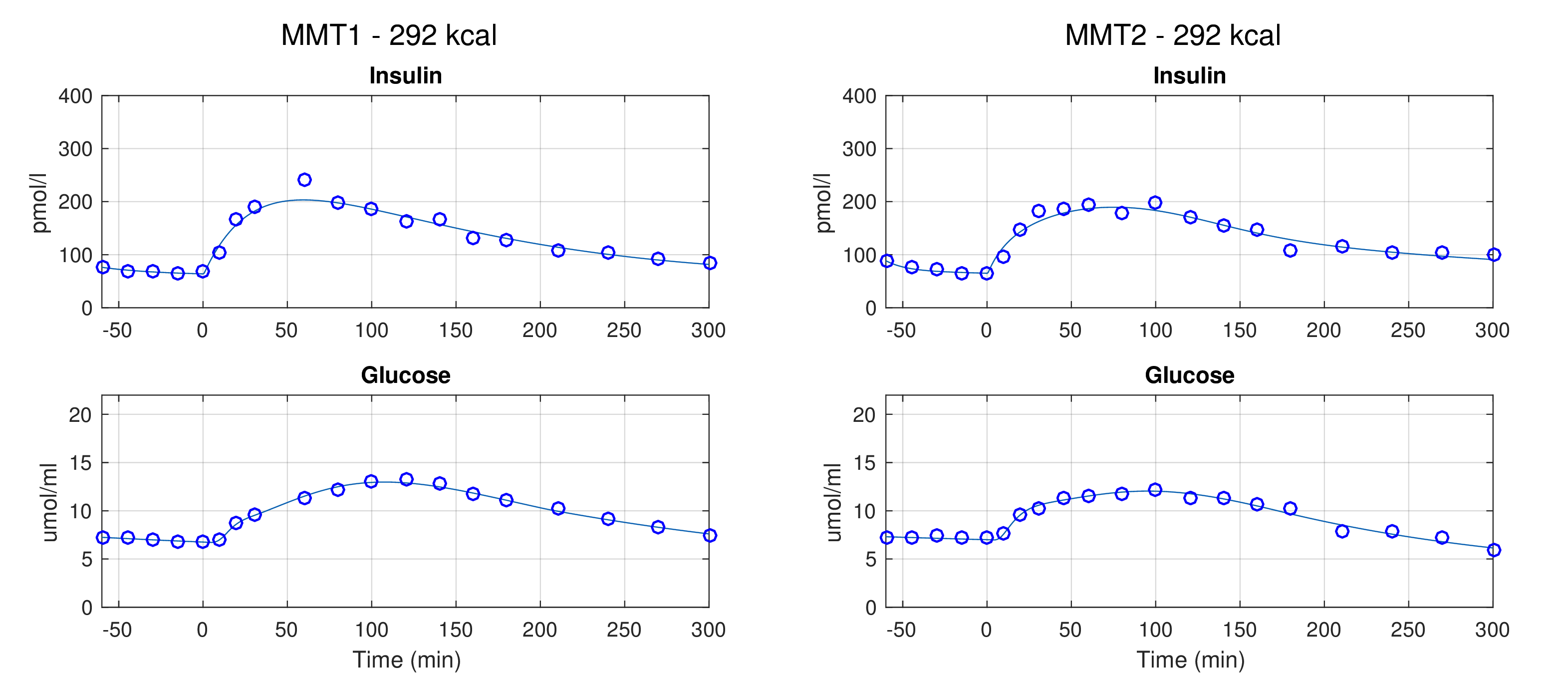


**Figure S3 - Simulation outputs of the GLUKINSLOOP 2.0 (patient 2).** The figure shows the time courses of plasma insulin and glucose concentrations during the MMT1 (left panel) and MMT2 (right panel) in the second study participant. Experimental data are shown as blue dots, while the simulated time courses are provided as a continuous blue line.


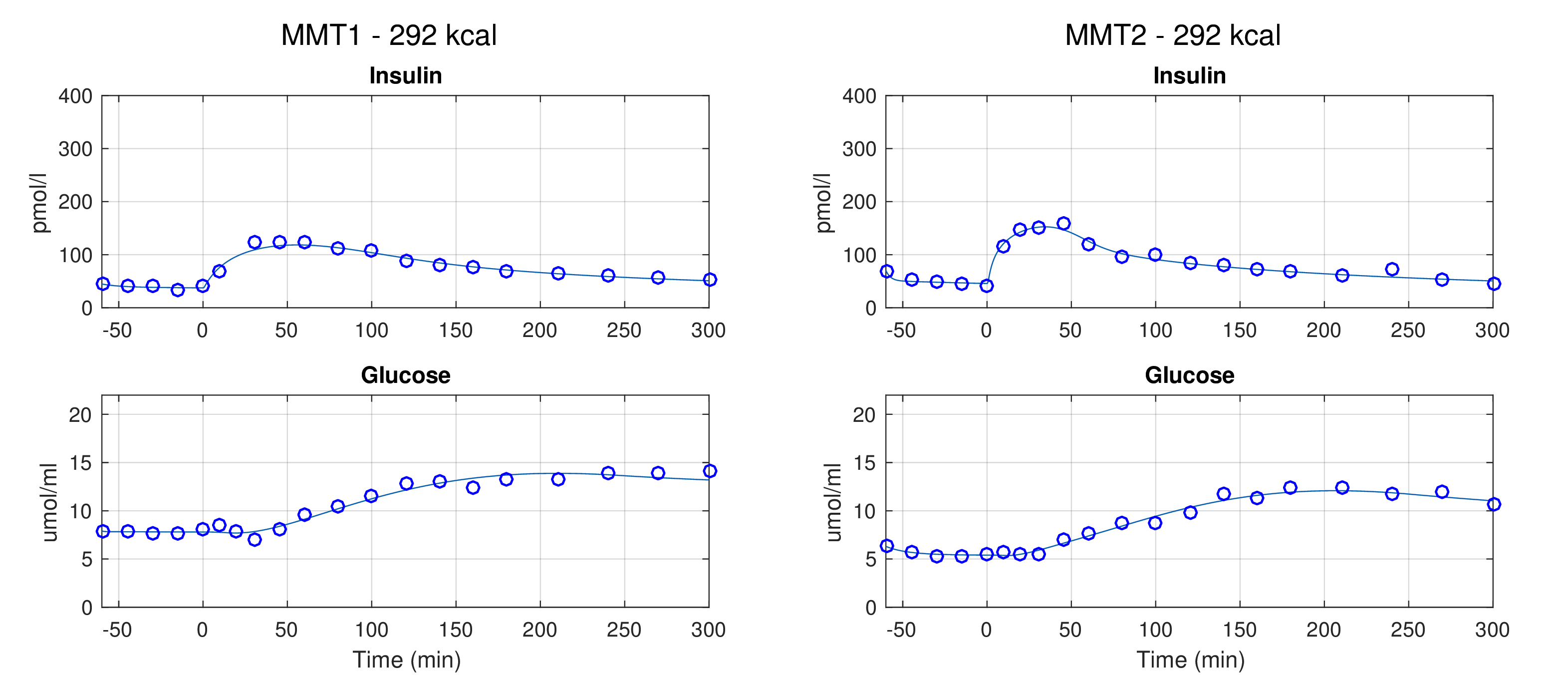


**Figure S4- Simulation outputs of the GLUKINSLOOP 2.0 (patient 3).** The figure shows the time courses of plasma insulin and glucose concentrations during the MMT1 (left panel) and MMT2 (right panel) in the third study participant. Experimental data are shown as blue dots, while the simulated time courses are provided as a continuous blue line.


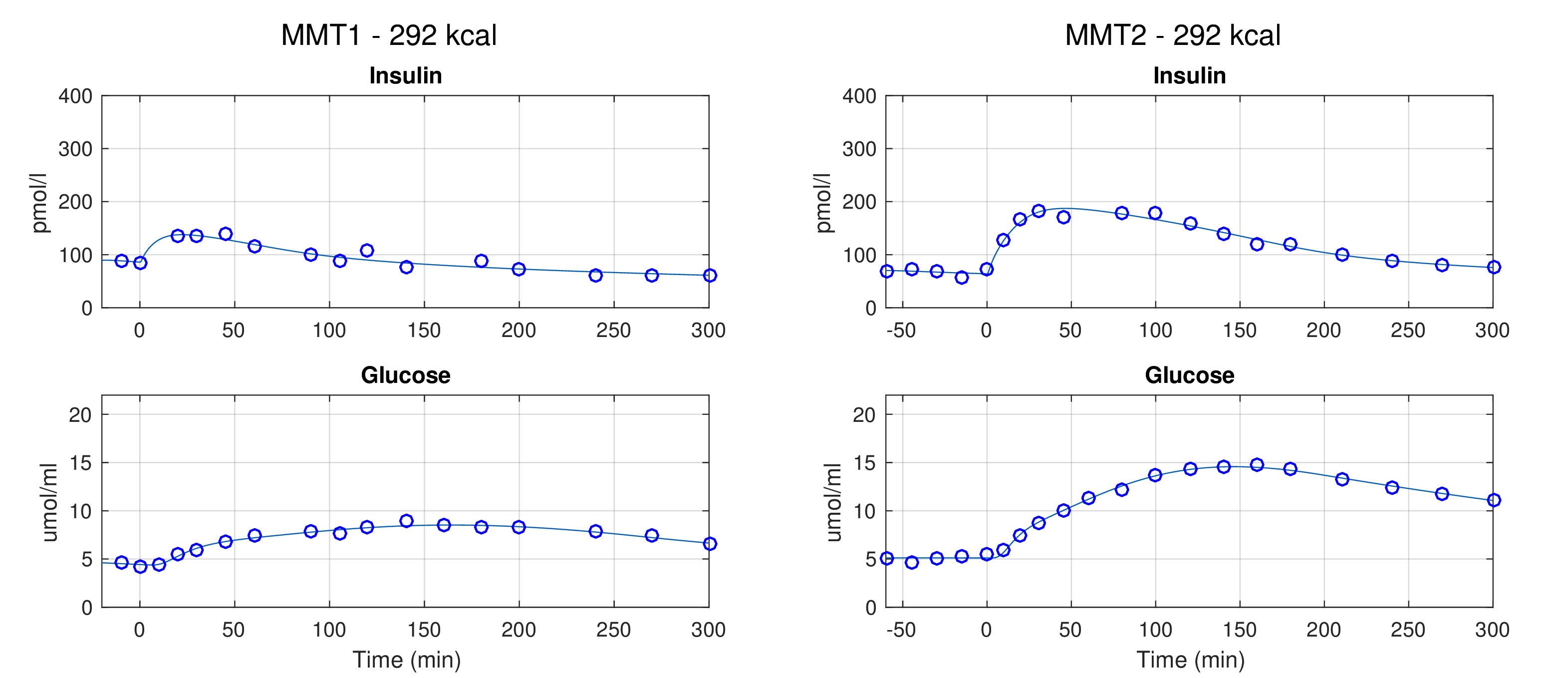


**Figure S5 - Simulation outputs of the GLUKINSLOOP 2.0 (patient 4).** The figure shows the time courses of plasma insulin and glucose concentrations during the MMT1 (left panel) and MMT2 (right panel) in the fourth study participant. Experimental data are shown as blue dots, while the simulated time courses are provided as a continuous blue line.


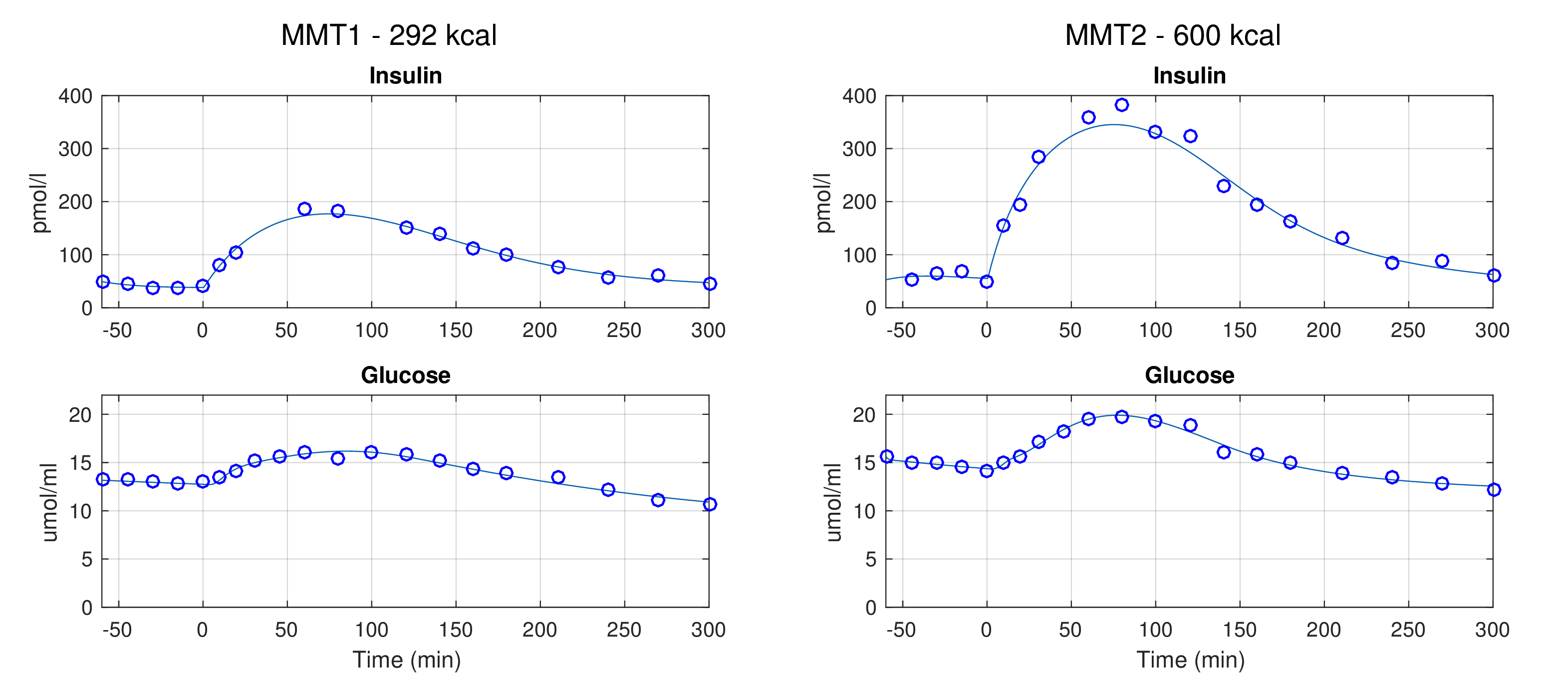


**Figure S6 - Simulation outputs of the GLUKINSLOOP 2.0 (patient 5).** The figure shows the time courses of plasma insulin and glucose concentrations during the MMT1 (left panel) and MMT2 (right panel) in the fifth study participant. Experimental data are shown as blue dots, while the simulated time courses are provided as a continuous blue line.


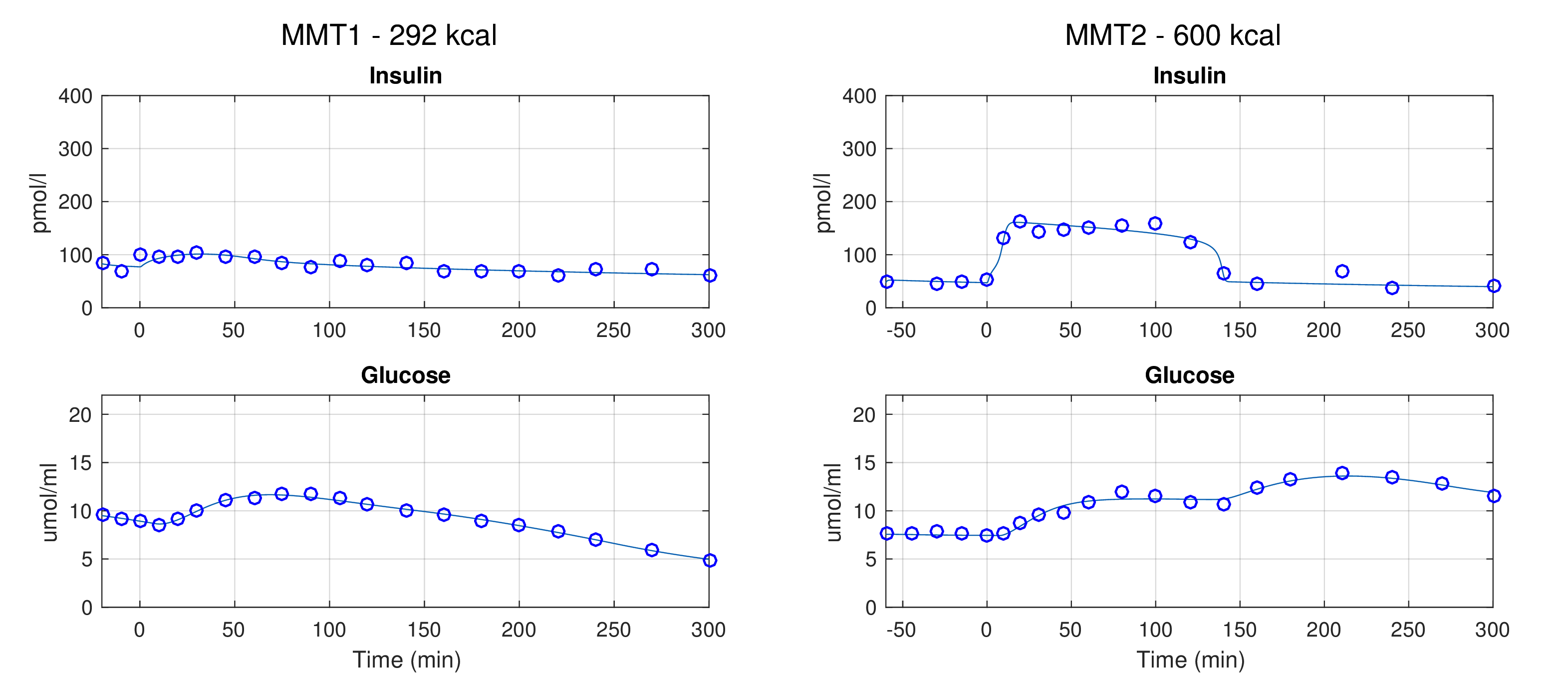


**Figure S7 - Simulation outputs of the GLUKINSLOOP 2.0 (patient 6).** The figure shows the time courses of plasma insulin and glucose concentrations during the MMT1 (left panel) and MMT2 (right panel) in the sixth study participant. Experimental data are shown as blue dots, while the simulated time courses are provided as a continuous blue line.


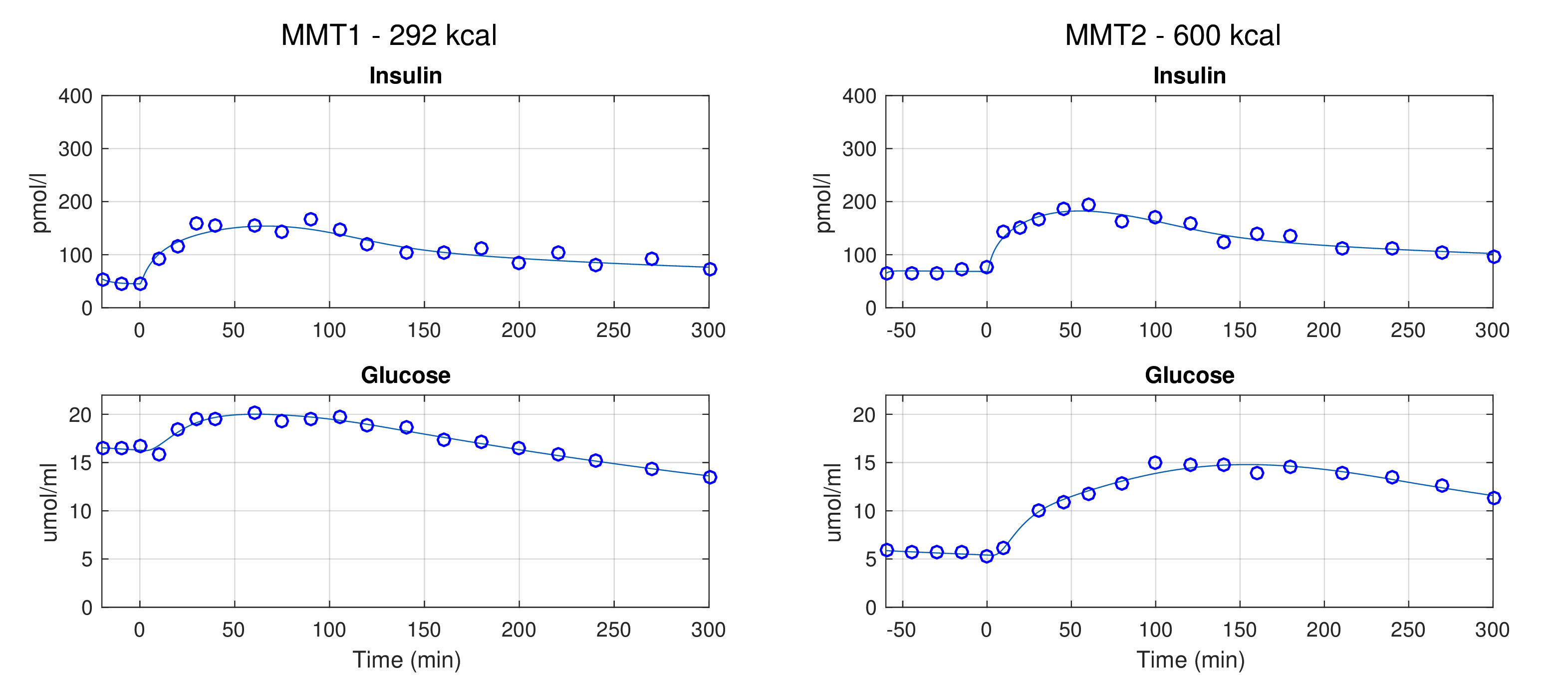


**Figure S8 - Simulation outputs of the GLUKINSLOOP 2.0 (patient 7).** The figure shows the time courses of plasma insulin and glucose concentrations during the MMT1 in the seventh study participant. Experimental data are shown as blue dots, while the simulated time courses are provided as a continuous blue line.


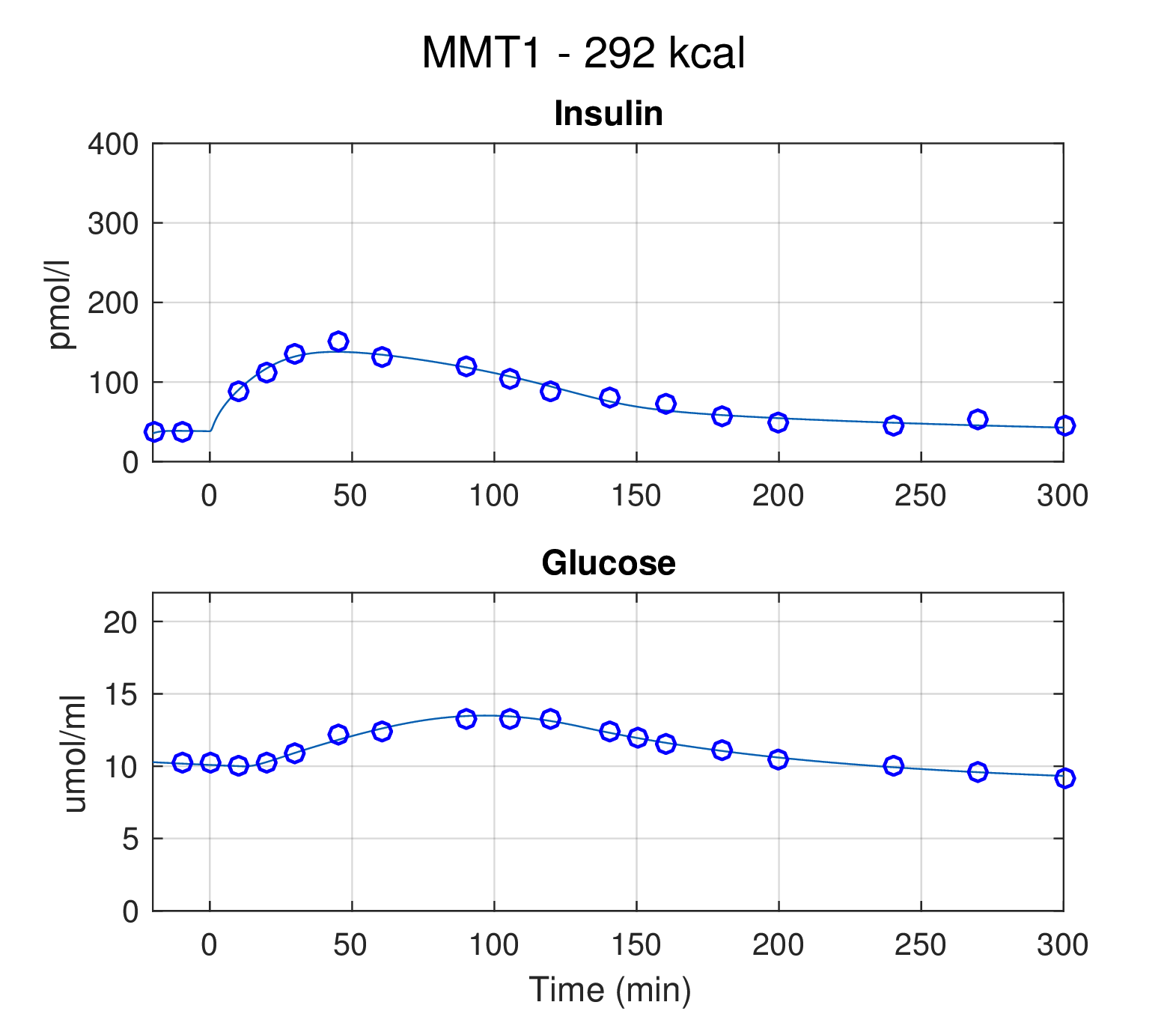


**Figure S9 - Simulation outputs of the GLUKINSLOOP 2.0 (patient 8).** The figure shows the time courses of plasma insulin and glucose concentrations during the MMT1 in the eighth study participant. Experimental data are shown as blue dots, while the simulated time courses are provided as a continuous blue line.


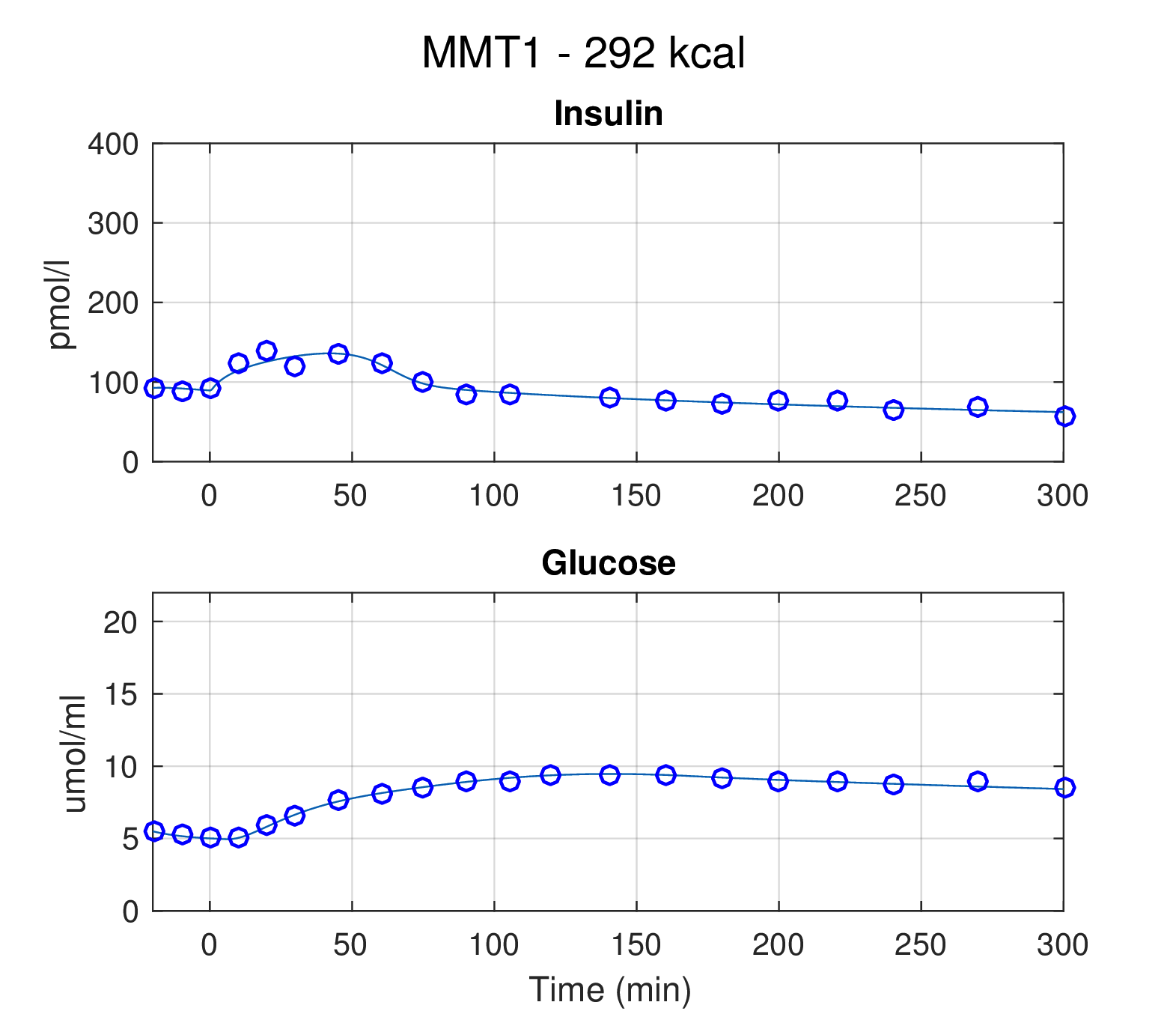


**Figure S10 - Simulation outputs of the GLUKINSLOOP 2.0 (patient 9).** The figure shows the time courses of plasma insulin and glucose concentrations during the MMT1 in the ninth study participant. Experimental data are shown as blue dots, while the simulated time courses are provided as a continuous blue line.


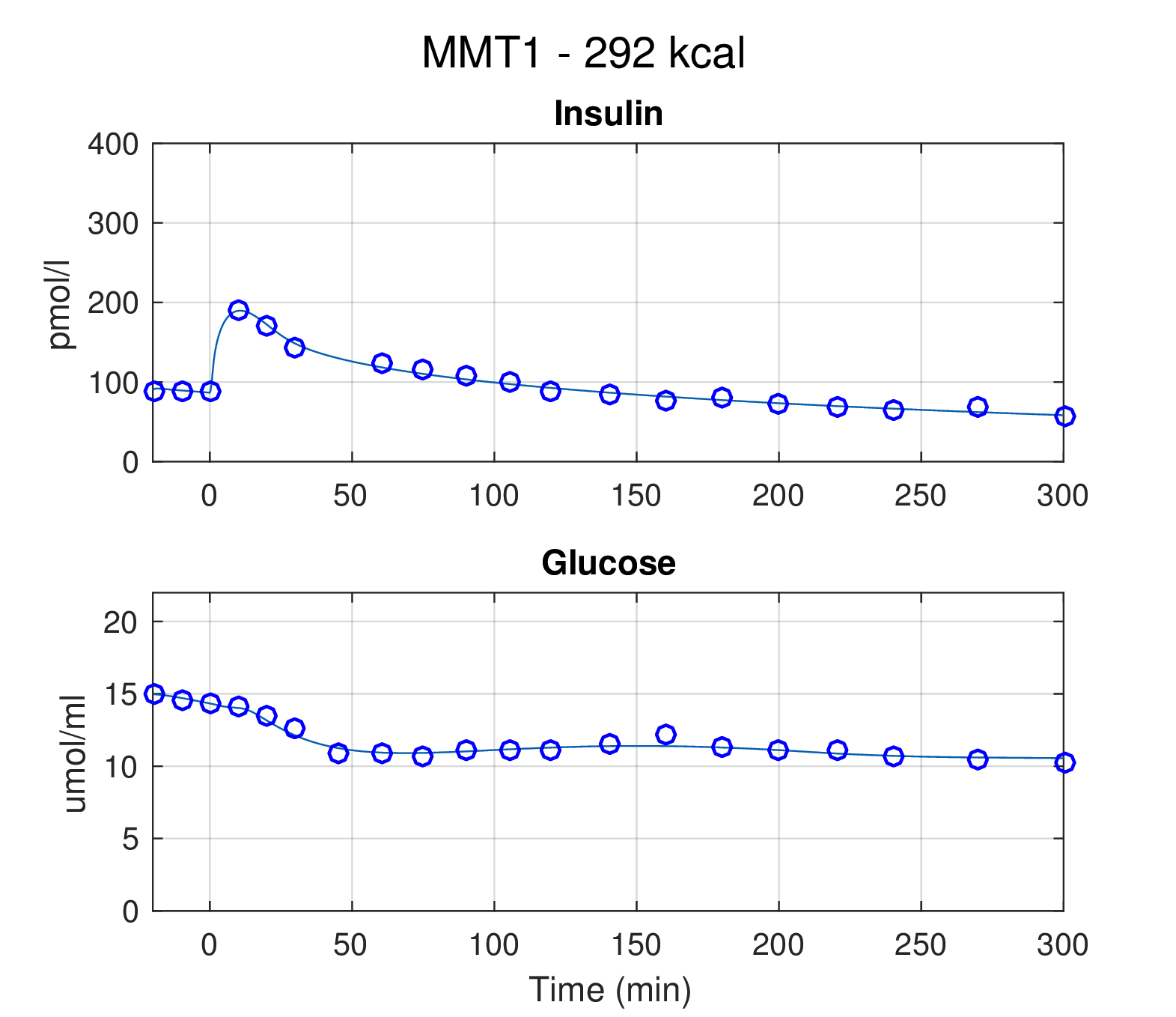


**Figure S11 - Simulation outputs of the GLUKINSLOOP 2.0 (patient 10).** The figure shows the time courses of plasma insulin and glucose concentrations during the MMT1 in the tenth study participant. Experimental data are shown as blue dots, while the simulated time courses are provided as a continuous blue line.


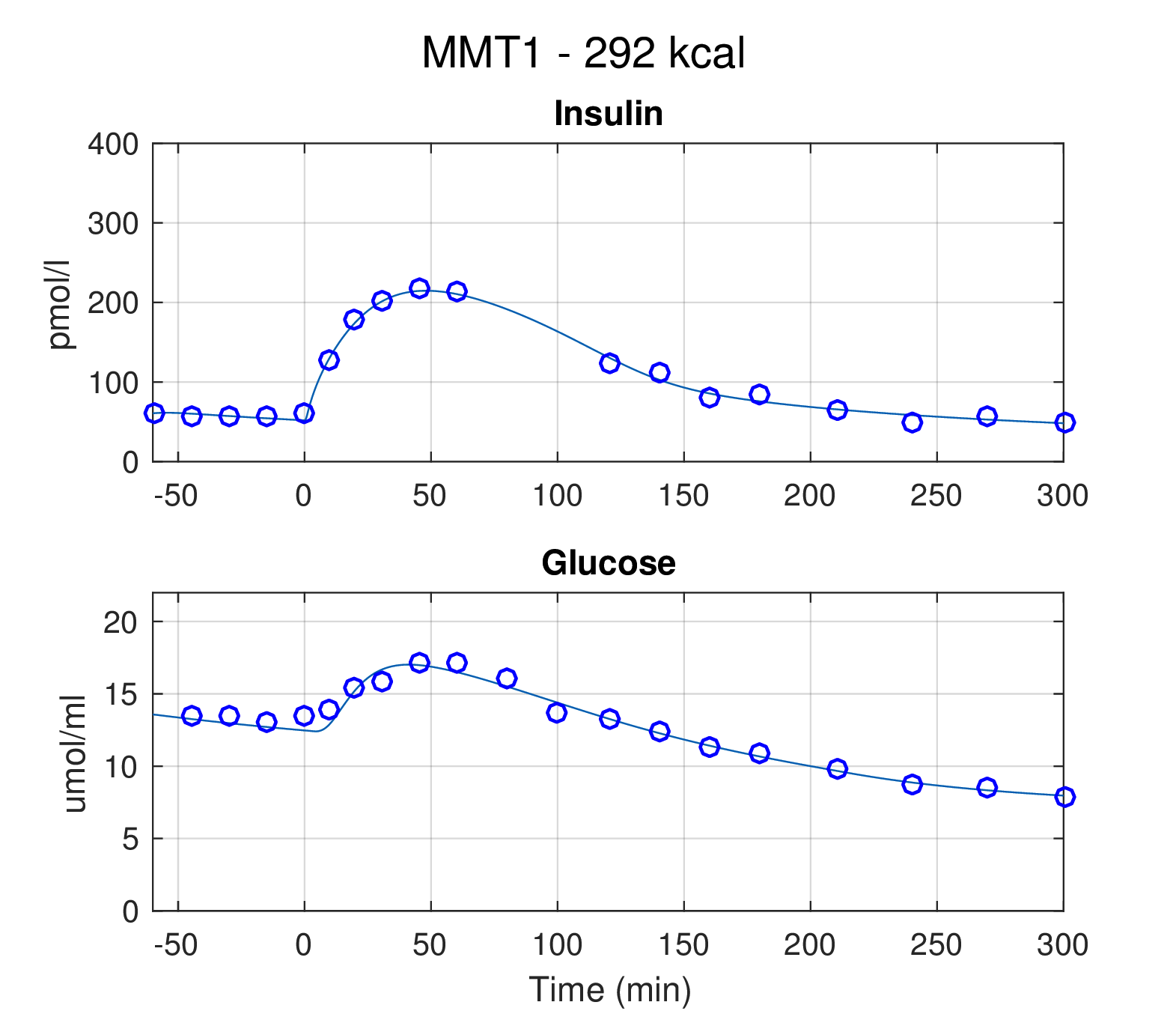


**Figure S12 – The Oral Glucose Input function (OGI).** The OGI function describes the rate of appearance of glucose that reaches the bloodstream after oral ingestion. The plots depict mean±SEM of the OGI functions (expressed in μmol/min) during the different MMTs.

**1.4 References**

1 Polonsky, K. S. *et al.* Quantitative study of insulin secretion and clearance in normal and obese subjects. *J Clin Invest* **81**, 435-441, doi:10.1172/JCI113338 (1988).

2 Piccinini, F., Dalla Man, C., Vella, A. & Cobelli, C. A Model for the Estimation of Hepatic Insulin Extraction After a Meal. *IEEE Trans Biomed Eng*, doi:10.1109/TBME.2015.2505507 (2015).

3 Toffolo, G., Campioni, M., Basu, R., Rizza, R. A. & Cobelli, C. A minimal model of insulin secretion and kinetics to assess hepatic insulin extraction. *Am J Physiol Endocrinol Metab* **290**, E169-E176, doi:10.1152/ajpendo.00473.2004 (2006).

4 Morishima, T., Bradshaw, C. & Radziuk, J. Measurement using tracers of steady-state turnover and metabolic clearance of insulin in dogs. *The American journal of physiology* **248**, E203-208 (1985).

5 De Gaetano, A., Panunzi S, Matone A, Samson A, Vrbikova J, Bendlova B, et al. Routine OGTT: A Robust Model Including Incretin Effect for Precise Identification of Insulin Sensitivity and Secretion in a Single Individual. *PLoS ONE* **8**, doi:doi:10.1371/journal.pone.0070875 (2013).

6 Vella, A. *et al.* Type I diabetes mellitus does not alter initial splanchnic glucose extraction or hepatic UDP-glucose flux during enteral glucose administration. *Diabetologia* **44**, 729-737, doi:10.1007/s001250051682 (2001).

7 DeFronzo, R. A., Tobin, J. D. & Andres, R. Glucose clamp technique: a method for quantifying insulin secretion and resistance. *The American journal of physiology* **237**, E214-223 (1979).

8 Dalla Man, C. *et al.* Measurement of selective effect of insulin on glucose disposal from labeled glucose oral test minimal model. *Am J Physiol Endocrinol Metab* **289**, E909-914, doi:10.1152/ajpendo.00299.2004 (2005).
